# Advantages of an easy-to-use DNA extraction method for minimal-destructive analysis of collection specimens

**DOI:** 10.1371/journal.pone.0235222

**Published:** 2020-07-08

**Authors:** Franziska Patzold, Alberto Zilli, Anna K. Hundsdoerfer

**Affiliations:** 1 Museum of Zoology (Museum für Tierkunde), Senckenberg Natural History Collections Dresden, Dresden, Germany; 2 Division Insects, Department Life Sciences, Natural History Museum, London, United Kingdom; Tierarztliche Hochschule Hannover, GERMANY

## Abstract

Here we present and justify an approach for minimal-destructive DNA extraction from historic insect specimens for next generation sequencing applications. An increasing number of studies use insects from museum collections for biodiversity research. However, the availability of specimens for molecular analyses has been limited by the degraded nature of the DNA gained from century-old museum material and the consumptive nature of most DNA extraction procedures. The method described in this manuscript enabled us to successfully extract DNA from specimens as old as 241 years using a minimal-destructive approach. The direct comparison of the DNeasy extraction Kit and the Monarch® PCR & DNA Clean-up Kit showed a significant increase of 17.3-fold higher DNA yield extracted with the Monarch Oligo protocol on average. By using an extraction protocol originally designed for oligonucleotide clean-up, we were able to combine overcoming the restrictions by target fragment size and strand state, with minimising time consumption and labour-intensity. The type specimens used for the minimal-destructive DNA extraction exhibited no significant external change or post-extraction damage, while sufficient DNA was retrieved for analyses.

## Introduction

Insects are the most diverse animal group on earth, with more than 1 million described species and an unknown number of undescribed species [1, 2]. Within the last years, an increasing number of genetic studies used insects from museum collections for purposes of phylogenetic inference [2, 3], biogeography studies [4], and taxonomic identifications [5], adding to our understanding of biodiversity [6–10].

However, the availability of specimens for molecular analyses has been limited by two main factors:

- Most museum specimens are not preserved to keep their DNA from degrading and inter-strand cross-linking. Thus, most successful DNA extractions are restricted to recently collected specimens and those preserved specifically for molecular work [2, 11–13]. This excludes many insect species, which are only known from a few (even just one) individuals, which were collected many years ago and have just been traditionally preserved in dry condition [2].
- The consumptive nature of the DNA extraction procedures created a conflict between DNA studies and the archival maintenance of the collections [14], which is particularly true for small specimens, such as insects, where even limited sampling is likely to destroy important morphological characters [15].

Degradation of DNA into smaller fragments is caused by several factors including exposure to radiation (mainly UV), temperature, pH, chemicals used for specimen preservation, and free water, resulting in an inverse correlation between sample age and fragment length [2, 13, 16–21]. These factors drive single-strand nicks and double-strand breaks, which leads to an accumulation of highly fragmented DNA and single-stranded DNA [1, 13, 22]. Therefore, both highly fragmented DNA and single-stranded DNA (ssDNA) need to be targeted in DNA extraction from museum specimens. Customised non-commercial protocols (non-kit) were shown to be successful in DNA extraction from subfossil and museum specimens [23]. However, they are very time consuming and thus not suitable for high throughput next generation sequencing (NGS) approaches. The transition of these protocols to a silica column-based approach would help to overcome this restriction and drive the study of century-old museum samples.

The conflict between the maintenance of collections and the usage of specimen DNA for research could be solved by the application of an extraction protocol designed to recover DNA from insect body parts without bringing externally visible morphological damage to the material [2]. Such protocols were shown to be successful in beetles [2, 15], but to date, no similar protocol was developed for Lepidoptera as their dense vestiture of scales adds complexity to non-destructive DNA isolation approaches.

Here, we report the results of a study that tested the potential of obtaining historic insect DNA for NGS of museum specimens by comparing three different silica-column-based kits. To enable comparisons, all of these were used in a destructive approach (ground insect legs). Additionally, the application of one of these protocols to a minimal-destructive extraction of DNA from type specimens dating back to 1779 (legs left intact) was tested. The latter approach holds the potential of obtaining historic insect DNA without the destruction of valuable specimens and their morphological traits, as well as providing data on the genetic constitution of populations that may no longer exist.

## Material and methods

DNA was extracted from one leg, each of a total of 230 archived hawkmoth specimens (17 species of the genus *Hyles*), which were collected between 1779 and 2017, including five type specimens. The samples were obtained from 17 museums and natural history collections (S1 Table). None of the specimens were initially preserved foreseeing any DNA extraction but rather to maintain their habitus and morphology. All specimens were labelled with MTD-TW (Museum für Tierkunde Dresden–Tissue “Wirbellose”) catalogue numbers of the Molecular Biology Laboratory of the Natural History Collections Senckenberg Dresden, and an individual collection number. DNA was extracted from 114 specimens using the DNeasy extraction Kit (Qiagen), while DNA from 35 specimens was extracted using the innuPREP DNA Mini Kit (Analytik Jena), in both cases following the manufacturer manuals. The DNA of the remaining 82 samples was extracted using the Oligonucleotide Clean-up protocol of the Monarch® PCR & DNA Clean-up Kit (New England Biolabs). Furthermore, we used two legs from six of these specimens to extract DNA with both the Monarch® PCR & DNA Clean-up Kit and the DNeasy extraction Kit for a direct comparison of the extraction methods. The legs were not cleaned by UV-light or chlorine solution before extraction. Such handling is typical for the extraction of DNA from bones and teeth, but due to the less resistant chitinous exoskeleton of Lepidoptera, it would have led to full degradation of the DNA inside the legs and to destruction of both the structure and vestiture of scales. Full precautions were taken for all 230 extractions to prevent contaminating the samples with previously extracted or amplified DNA. The extractions of the specimens older than ten years were conducted in a laboratory dedicated to research on samples that contain low amounts of degraded DNA from historic museum or subfossil specimens (“ancient DNA”, aDNA, see also [24], Box I). This laboratory is physically isolated from the laboratory where PCR and post-PCR work is performed. The bench was decontaminated before handling of the specimens by using UV-light and DNA AWAY (ThermoFisher Scientific). Forceps were cleaned with DNA AWAY before handling specimens. Only guaranteed DNA-free disposable consumables were used, and reagents were dedicated to use in these protocols of aDNA research only. Filter tips were used for all experimental procedures.

Two approaches in tissue handling and DNA extraction are compared:

- the common leg grinding with three different silica-column-based kits, including DNeasy extraction Kit (Qiagen), innuPREP DNA Mini Kit (Analytik Jena) and Monarch® PCR & DNA Clean-up Kit (New England Biolabs);
- and the minimal-destructive usage of the Monarch® PCR & DNA Clean-up Kit (New England Biolabs), where legs of the type specimens were left intact.

### Destructive sampling of non-type specimens

The legs of the 148 specimens studied using the DNeasy extraction Kit (Qiagen) or the innuPREP DNA Mini Kit (Analytik Jena) were placed in 1.5 ml microcentrifuge tubes and ground using an oscillating mill (MM 400, Retsch). Legs were ground for 1 min at a frequency of 25 Hz, using two sterile stainless-steel balls with a diameter of 3 mm. Lysis and DNA extraction was performed following the manufacturer protocol, with some modifications. This included incubation of lysis overnight at 56°C, and elution in a two-step procedure to maximise DNA yield, where each step was carried out using 35 μl elution buffer and incubation for 10 min, resulting in a total volume of 70 μl.

Additional legs of 77 samples were used with the Monarch® PCR & DNA Clean-up Kit (New England Biolabs), and ground as described above. The lysis was performed by using 45 μl of lysis buffer FN (AGOWA) and 5 μl proteinase K (Analytik Jena). All lysis reactions were incubated overnight at 56°C on a Mixing Block (MB-102, Thermocell). Mixing speed was set to 450 rpm (ground legs). Extraction was performed following the Oligonucleotide Clean-up protocol of the Monarch® PCR & DNA Clean-up Kit (New England Biolabs), using 100 μl DNA Clean-up Binding Buffer, 300 μl ethanol (≥ 95%) and 500 μl DNA Wash Buffer. The washing step was performed twice to minimise impurities. Elution was performed in a two-step procedure with each step using 15μl. The protocol can be found at dx.doi.org/10.17504/protocols.io.8m9hu96.

### Minimal-destructive sampling of type specimens

In contrast, the legs of the five type specimens remained mechanically undamaged and were glued back to the specimen after lysis. The lysis was performed by using 135 μl of lysis buffer FN (AGOWA) and 15 μl proteinase K (Analytik Jena). All lysis reactions were incubated overnight at 56°C on a Mixing Block (MB-102, Thermocell), mixing speed was set to 300 rpm. Extraction was performed following the Oligonucleotide Clean-up protocol of the Monarch® PCR & DNA Clean-up Kit (New England Biolabs) as described above.

### Measurements, imaging and statistics

DNA concentration was measured using the Qubit Fluorometer high sensitivity assay (ThermoFisher), and fragment length were assessed with 4200 TapeStation using the High Sensitivity D1000 ScreenTape (Agilent). The total amount of extracted DNA was calculated by the measured DNA concentration multiplied by the volume of elution buffer used. Photos of moths were taken with a Sony α6300 and a Sony E 16–50 mm F3.5–5.6 OSS, or with a Huawei CLT-L29. Correlation analysis and plot graphics were done using R, RStudio, ggplot2 and ggpubr.

### Shotgun library preparation and sequencing

To test the integrity of the extracted DNA from the Monarch Oligo extraction protocol, we performed a full NGS analysis, including bioinformatics after sequencing.

Whole genome shotgun libraries were prepared from 10 museum specimens with DNA extracted using the Monarch® PCR & DNA Clean-up Kit, based on a published protocol for degraded DNA samples [25], which was modified in order to incorporate adapter design that enables high sample multiplexing on a single sequencing lane [26–28]. They were sequenced on a 75 bp paired-end Illumina MiSeq run. Percentages of duplicates and adaptors were estimated using FastQC [29]. Removal of low-quality reads and adaptor sequences (Illumina) was performed with BBDuk from BBTools [30].

The origins of these sequences were tested with Kraken2, a taxonomic classification system based on k-mer matches to achieve high accuracy in the classification of sequences. It matches each k-mer within a query sequence to the lowest common ancestor (LCA) of all genomes containing the given k-mer. The k-mer assignments inform the classification algorithm [31]. The standard database of Kraken2 was expanded with a reference genome and mitochondrial genome of the genus *Hyles*, consisting of assembled long-reads from a single *Hyles vespertilio* specimen [32].

### Multi sequence alignment and phylogenetic tree

The quality controlled and trimmed reads of the ten individuals were mapped both on the nuclear and the mitochondrial reference genome of the genus *Hyles* using the local Aligner of the NextGenMap [33]. Afterwards, the alignment was filtered for unambiguous mapped reads (MQ < 10) using Samtools [34], and duplicate reads were removed using Picard Tools (http://broadinstitute.github.io/picard). Coverage of each alignment was conducted using bedtools [35] and custom scripts. To validate the alignments on the mitochondrial genome, a two-step approach was used. First, the gene fragments of the cytochrome oxidase subunits COI and COII plus the intermediate tRNA^Leu^ (trnL2) were annotated using MITOS [36]. In a second step sanger sequences of COI, COII and trnL2 sequences of the same or closely related species from an earlier publication [37] were mapped on the mitochondrial reference genome to validate the annotated positions of each gene. These sequences are available on their NCBI accession numbers (see Fig 8). After that, the consensus sequences from the alignments were called at the confirmed positions of the genes using Samtools and BCFtools [38]. Positions in the alignments with zero coverage were masked out with “N”. The consensus sequences were used together with sanger sequences from previous publications [37] to enable a multi sequence alignment, which was done using MAFFT [39], allowing adjusting of sequence direction according to the first sequence. To control for a possible reference bias in the phylogenetic tree reconstruction, the sequence of the mitochondrial reference genome was also included in the multi sequence alignment. This alignment (43 sequences in total) was subsequently used to define codon positions for the protein-coding genes COI and COII. Codons were known from the MITOS alignment. These codon positions were used in PartitionFinder2 [40]. We used the greedy search algorithm and PhyML to define most likely partitions and best-fitting models of these in the alignment [41, 42]. For the generation of a phylogenetic tree, we used these partitions and the best fit model for each of these in IQ-Tree [43]. Since our ingroup are Palaearctic *Hyles*, we chose to include New World *Hyles perkinsi* (FN386584) and *Hyles calida* (AJ749426, Hawaii) as outgroup sequences to this reconstruction.

## Results and discussion

### DNA Extraction from destructive sampled non-type specimens

The merit of museum material in molecular studies for biological research is often limited by the initial step of DNA extraction, as DNA from such specimens is naturally highly fragmented leading to frequently very low amounts of DNA that can be extracted [44]. To understand if the Oligonucleotide Clean-up protocol of the Monarch® PCR & DNA Clean-up Kit enables the extraction of higher amounts of DNA, we compared this kit with two commonly used kits for the DNA extraction from museum specimens. For a better comparison between the kits, only measurements of ground legs were included.

All three tested kits yielded good amounts of DNA within the tested age range. The DNeasy kit showed a higher maximum of extracted DNA in comparison to the Monarch kit, while the maximum of the innuPREP kit is lower.

Both kits yielded slightly lower mean amounts of DNA compared to the Monarch kit (Table 1).

**Table 1:** Overview of all three kits used with the destructive approach to compare effectiveness in DNA extraction.

Type of kit used	Mean sample age [years] (range)	Total DNA extracted [ng], mean (range)
DNeasy extraction Kit	20.2 (3–54)	259.4 (0.0–1820.0)
innuPREP DNA Mini Kit	13.9 (2–25)	264.4 (0.0–1010.0)
Monarch PCR & DNA Clean-up Kit	28.8 (3–112)	316.5 (33.1–1106.0)

**Fig 1:**
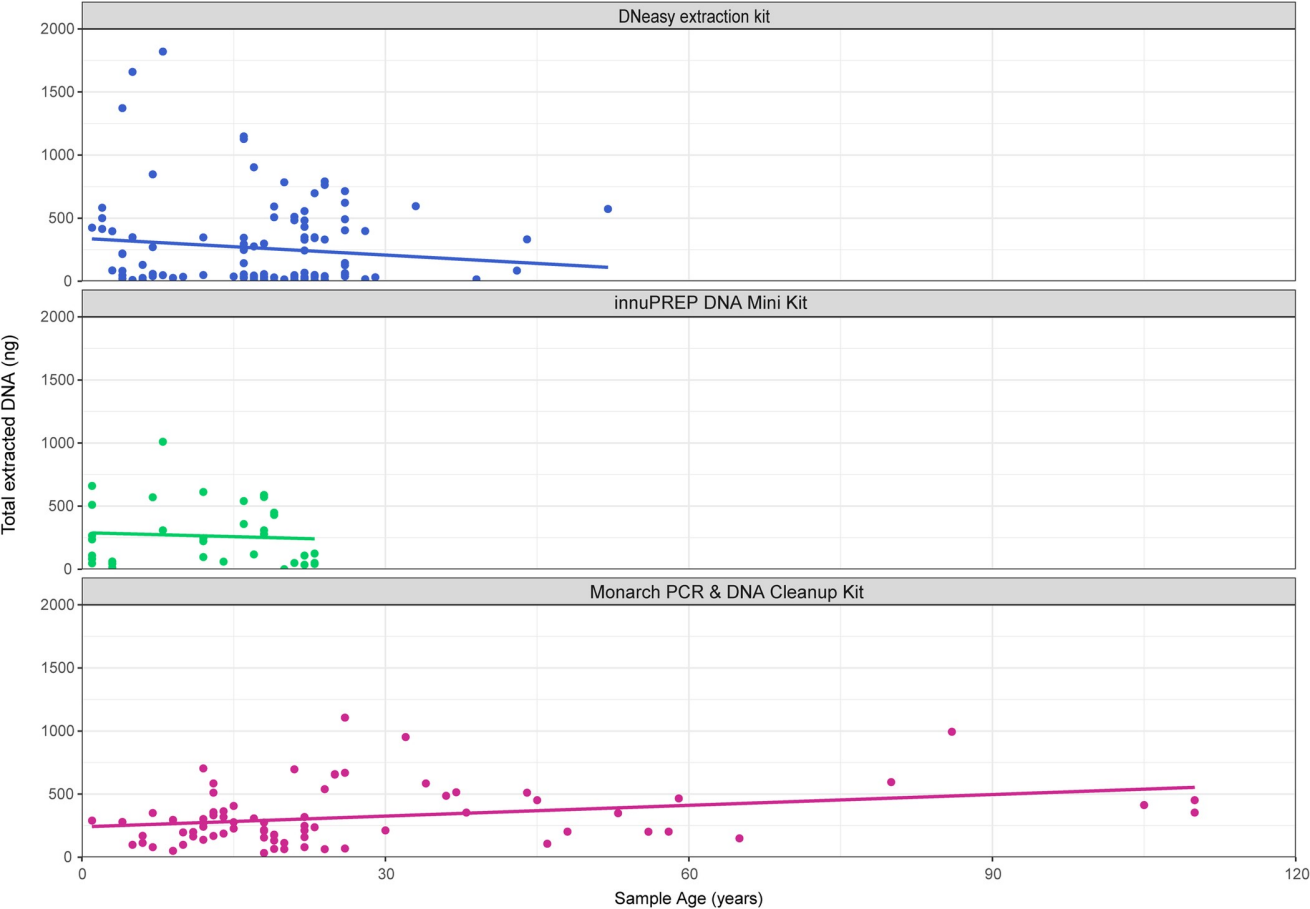
Distribution of the total extracted DNA from the three kits. (A) samples extracted with the DNeasy extraction Kit (Qiagen); dots in green are the samples extracted using the innuPREP DNA Mini Kit (Analytik Jena), and the dots in magenta are samples extracted with the Monarch® PCR & DNA Clean-up Kit (New England Biolabs). A trend line was included for each protocol for visualisation of overall distribution.

The DNA extraction using the DNeasy and the innuPREP kits failed on three individuals in total. From these, two failed using the DNeasy extraction kit (MTD-TW 11690 and MTD-TW 1723, 20 and 19 years of age), and one using the innuPREP extraction kit (MTD-TW 8965, 21 years old). None of the samples extracted with the Monarch Oligo extraction kit failed, despite the tested age range of this kit being clearly broader than in the samples tested with the other two kits (Table 1). This difference in maximal age of the tested samples is due to the need to limit destructive extractions from century-old museum specimens to as few as possible even in method testing. Extractions of these were limited to the Monarch Oligo extraction kit using intact legs, as the other two kits started to fail in measurable DNA yields from samples at about 20 years of age and older (Fig 1), although the amount of extracted DNA and the respective sampling year were not significantly correlated for all three kits tested (DNeasy p = 0.217, innuPREP p = 0.689, Monarch p = 0.054).

Nevertheless, we found that the mean amount of extracted DNA with the Monarch® PCR & DNA Clean-up kit increases slightly with the age of samples (Fig 1C), while the contrary effect was visible in the extractions done with the DNeasy and the innuPREP kits, where the mean of the DNA-yield decreased rapidly with age (Fig 1A and 1B). These diverging results between the protocols were to be expected, since younger specimens contain a higher percentage of intact double-stranded DNA (dsDNA) [22], leading to more efficient DNA extraction with the DNeasy and the innuPREP kits, but less efficient DNA extraction by the Oligonucleotide Clean-up Protocol, as this favours the binding of ssDNA to the silica inside the column. Our findings are therefore similar to other studies where efficient DNA extraction from skeletal remains based on silica-columns was shown [45, 46].

Nevertheless, the DNA-yield is highly variable between specimens with all protocols (Fig 1). We expected this due to differing killing and post-mortem conditions of each individual, as the specimens’ originated from different museums and natural history collections, where insulation from ultraviolet light and stable room temperatures are not necessarily present in all collections [4]. This would be consistent with the results of other studies [17, 19, 20, 47–49], where the authors found that the immediate post-mortem treatment of specimens is particularly important in the preservation of amplifiable DNA rather than storage time. In another study, the authors were able to show a correlation between higher temperatures in more southern cities and the PCR success of the extracted DNA from museum specimens [4]. The authors postutated, from their personal observation of suboptimal specimen storage in several collections (open windows, no air-conditioning), that the higher storage temperatures induced severe DNA fragmentation. The cause for this correlation could also be induced by the co-correlation between storage temperatures and higher humidity in more southern regions [50], where humidity is likely causing accelerated fragmentation of the DNA because of upheld endogenous nuclease activity and hydrolytic damage [24, 51]. Besides environmental effects, the usage of chemicals in the collections [21] and catastrophes the historic specimens went through over time (e.g. storage in cellars whilst rockets bombing in London and Dresden, flooding of the early Innsbruck collections [52]) may lead to further effects on the amount and fragmentation of DNA. However, the potential influence of environmental and other specific effects varies between the collections and therefore, could not be addressed in this study.

**Fig 2:**
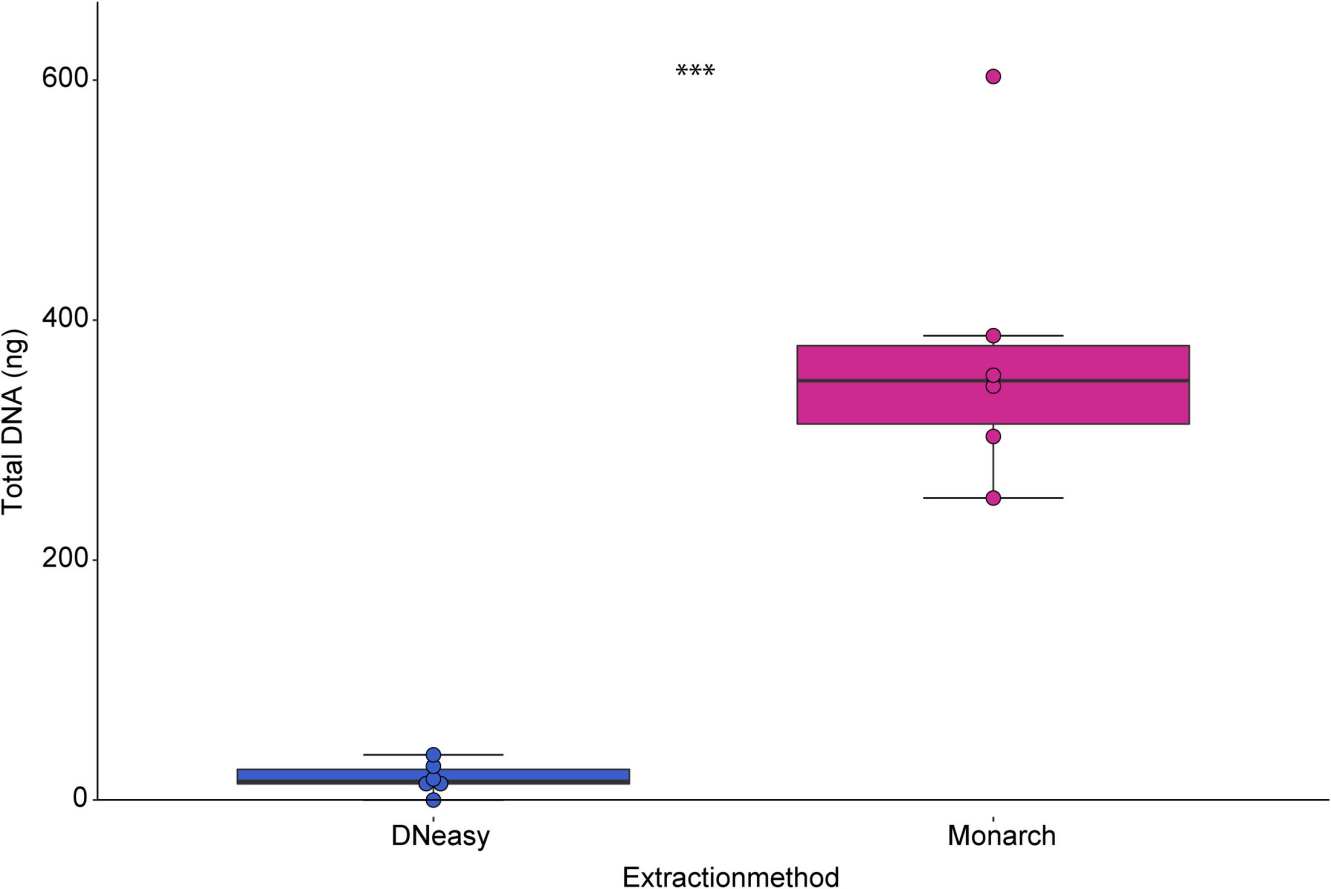
Comparison of total amounts of DNA extracted using the two protocols for the same individuals. The T-test was used to compare the means of the two protocols (*** p < 0.001).

The direct comparison of the DNeasy and the Monarch extraction kits (Fig 2 and S2 Table), carried out with one leg each from six specimens, which are between 13 and 137 years old, showed a significant increase of 355.5 ng in total DNA extracted, which equals to an average of 17.3-fold higher DNA yield from the Monarch kit compared to the DNeasy kit (p < 0.001).

This difference in extraction effectiveness (Figs 1 and 2) between the protocols appears to have two main reasons, which are the different cut-off sizes of the DNA fragments (100 bp in the DNeasy kit [53, 54]; 16 nt in the Monarch kit [55]) and the favouring of different DNA strand states [31]. The Oligonucleotide Clean-up protocol of the Monarch PCR & DNA Clean-up kit, as described by NEB, includes the addition of two volumes of ethanol (whole sample volume: ethanol, 1:2) in comparison to the DNeasy kit (whole sample volume: ethanol, 1:1), which shifts the binding DNA cut-off not only to shorter DNA fragments (from 25 nt down to 16 nt length) but also changes the binding efficiency of dsDNA (double-stranded DNA) to the membrane of the column, leading to a favoured extraction of ssDNA [31].

One of the six specimens used for the direct comparison of the differences in extraction effectiveness failed for DNA extraction with the DNeasy kit. At the same time, we extracted 603 ng from the same individual using the Monarch kit (Table 1 and Fig 2). As this specimen was only 13 years old (MTD-TW 9255), we would have expected good DNA-yield by using both kits. Similar results were described in another study where three one-year-old Lepidoptera specimens failed completely after being kept in a relaxing jar for several days by the collector [49]. Similar to our specimen, those specimens were in perfect condition with respect to external integrity. The authors also reported similar problems with DNA extraction/amplification from relaxed specimens. The authors suggested that relaxing enhances DNA degradation. Although such handling is not documented for our specimen, this evaluation is congruent with our finding that the Monarch Oligo kit effectively extracted short DNA fragments and ssDNA, while the DNeasy kit failed to extract highly fragmented DNA.

**Fig 3:**
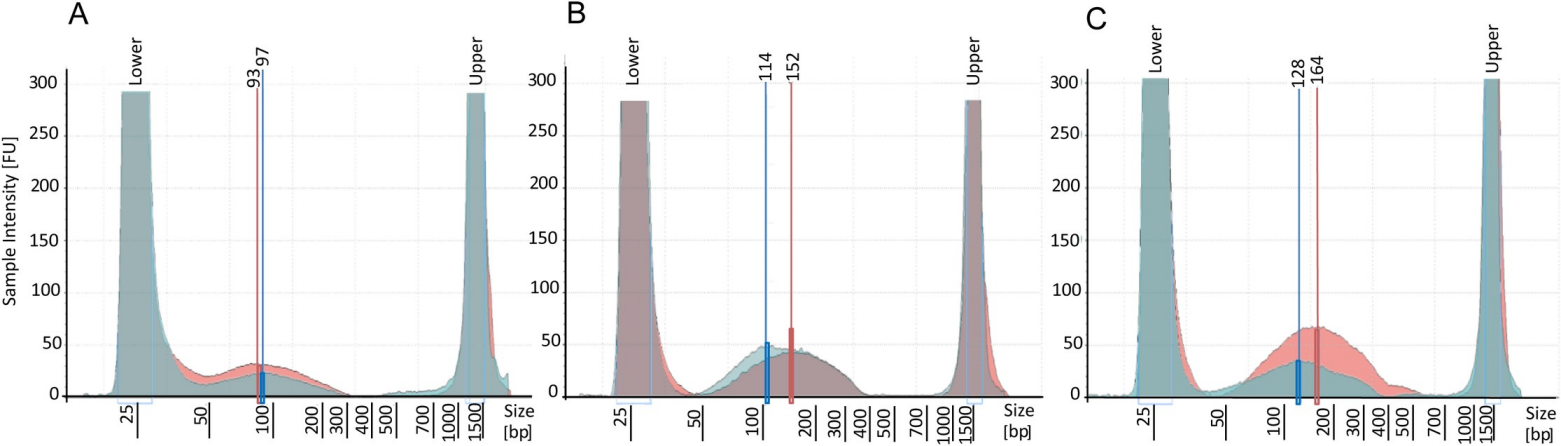
Comparison of fragment sizes between two protocols from three specimens. Electropherograms from the DNeasy extraction Kit (blue) and the Monarch PCR & DNA Clean-up Kit (red). The specimens individual MTD-TW numbers are as follows: A 9248, B 9252, C 9251. See Supplementary Table 1 for more details on the DNA yield from each extraction.

The change in the cut-off size was originally implemented in the oligonucleotide Clean-up kit to extract single-stranded oligonucleotides from a solution. Because other studies showed a significant correlation between the sample age and reduction in fragment length and strand state [14, 22, 46, 56–58], which causes an accumulation of fragmented ssDNA over time in century-old museum specimens [1, 13, 46], we transferred the application of the original Monarch Oligo protocol from the extraction of small oligonucleotides to the extraction of highly fragmented ssDNA. Contrary to our expectations, we did not observe a significant difference of fragment sizes in the direct comparison of the same specimen (Fig 3 and Table 2). Moreover, we encountered about the same fragment size distribution, with the mean yield of the Monarch extraction kit being slightly higher than those received from the DNeasy extraction kit (Table 2). Both extraction protocols recovered typical average molecule sizes (100–200 bp) which are expected in degraded DNA extracts [59].

**Table 2:** Fragment size distribution of two kits in comparison (ground legs).

Kit	Fragment size mean [bp] (range)	Peaks mean [bp] (range)
DNeasy	141 (50–637)	113 (97–130)
Monarch	154 (50–590)	137 (93–164)

Consequently, it appears likely that the shift in cut-off size did not lead to the expected extraction of significantly smaller DNA fragments, but rather merely to a shift in the targeted size range from 100 bp– 1 kb (DNeasy) to 16 bp– 500 bp (Monarch), which recovered the target fragment size necessary to build libraries for NGS of historical DNA [60] in both cases.

Customised non-commercial extraction protocols for ancient DNA, commonly advocated for museum samples, are also optimised for extraction of highly fragmented, ssDNA [23]. But these are mainly based on time-consuming phenol-chloroform extraction methods, which makes them not reasonably usable in high throughput experiments such as NGS approaches. By transferring the oligonucleotide Clean-up protocol for the extraction of highly fragmented DNA, we were able to overcome temporal restrictions underlying manual customised non-commercial protocols, similar to other silica spin column-based methods [46].

The preparation of Lepidoptera, especially of Sphingidae Macroglossini, is well documented in books of early Lepidopterists. Macroglossini used to be killed with a heated needle or hot water steam [61–63] until about 1900, and later with ethyl acetate, ethyl alcohol, cyanide or ammonia [64–66], depending on the collection method (e.g. light traps or hand collection), and the preference of the collector. These methods have varying influence on the preservation of the DNA as some, like the usage of hot water steam, are likely to denature the DNA of the specimen and may lead to prolonged drying periods which could lead to faster fragmentation of the DNA due to upheld endogenous nuclease activity and hydrolytic damage [24].

Furthermore, it is well known that Lepidoptera are often initially dried during fieldwork, and either pinned or stored in paper bags for years before wing-setting. This is done by rewetting with water vapour, with chemicals added against moulds (today mostly oxalic acid) in a jar to soften thoracic muscles and relax wings, and yet another drying [17, 49, 67]. This procedure has been shown to degrade DNA [2, 13, 16, 19, 49, 56]. The killing and post-mortem aspects of the individuals in our study were mostly undocumented and could be only assessed by reference to books of that time period; therefore, a correlation test for these circumstances and their influence on the DNA yield was not possible.

The workflow presented here used tissue material from hawkmoths (Sphingidae), which comprise some of the largest-sized Lepidoptera and therefore provide much tissue in one leg for DNA extraction. As the majority of Lepidoptera is smaller than hawkmoths usually are, the amount of extracted DNA might be correlated with the amount of tissue used for the extraction.

### Minimal-destructive DNA extraction from type specimens

As shown in the section above, the Oligonucleotide Clean-up protocol of the Monarch® PCR & DNA Clean-up Kit enabled us to extract higher amounts of DNA in old museum specimens (> 20 years). Yet the grinding of legs is not suitable for valuable type and century-old museum specimens. The necessity to preserve the external integrity of the specimens resulted in a minimal-destructive approach where legs were left intact and which was limited to the Monarch® PCR & DNA Clean-up Kit, as this was the only kit where none of the tested samples failed (see above).

The minimal-destructive DNA extraction procedure was tested on five type specimens, including three different species of the genus *Hyles*, collected between 1779 and 1789. Sufficient DNA for further analyses such as in an NGS approach was retrieved with a mean of 422.9 ng (range see Table 3), validating the use of this method on type specimens.

**Fig 4:**
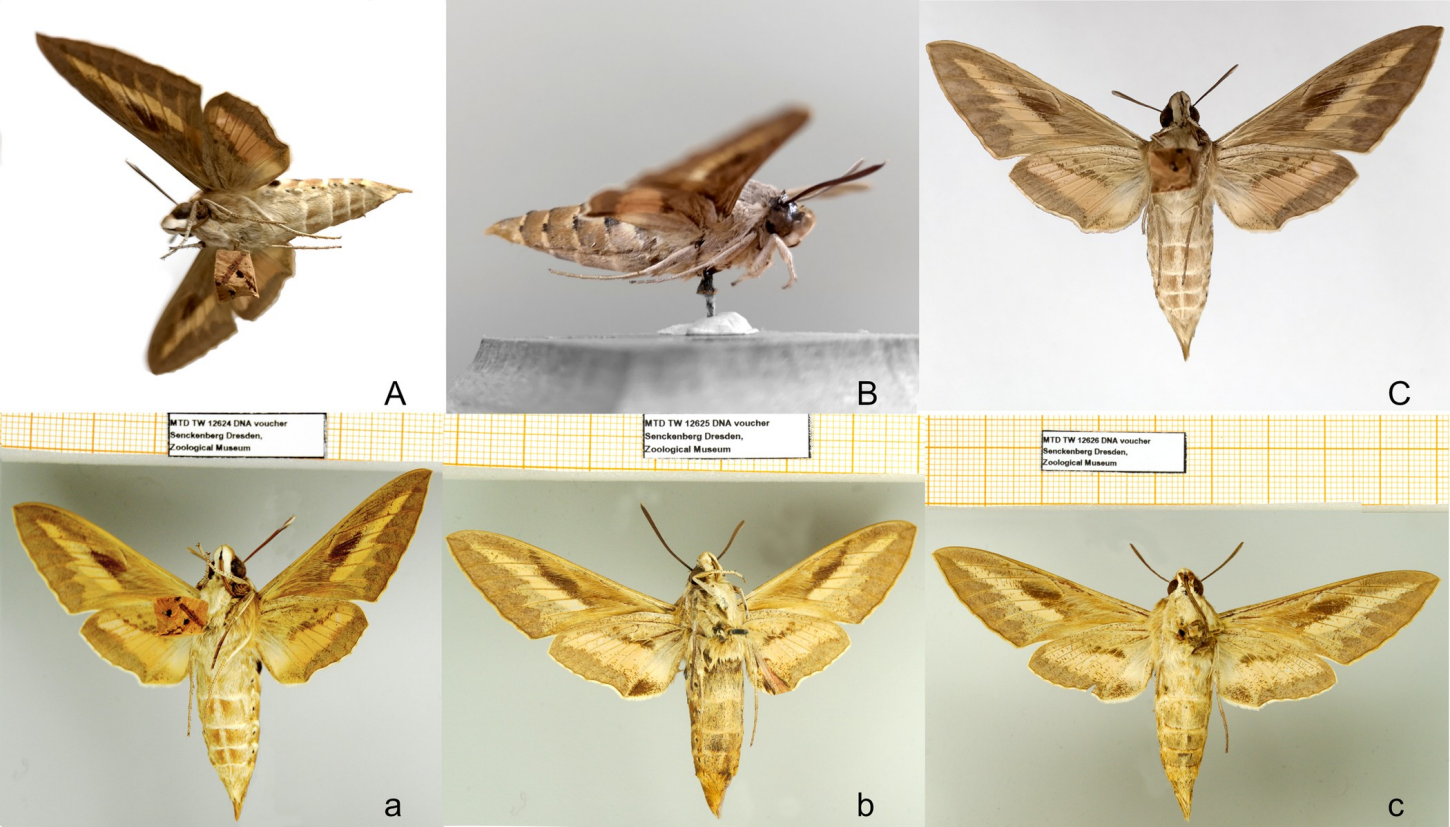
Lecto- and paralectotype specimens of *Hyles livornica* before (A, B, C) and after (a, b, c) sampling and extraction. Specimens were handled with cleaned forceps in the collection rooms. Treatment and reattachment of one leg each (middle leg). The specimens’ individual MTD-TW numbers are: A/a 12624, B/b 12625, C/c 12626 (S1 Table).

**Table 3:** Details of the five type specimens of *Hyles hippophaes* (Esper [1789]), *Hyles vespertilio* (Esper [1780]) and *Hyles livornica* (Esper [1780]) investigated in this study.

MTD-TW Accession No.	Species	Type Status	Location	Age (estimated)	Total DNA extracted (ng)
12622	*H*. *hippophaes*	Lectotype	Romania, Foran	[1789]	350.0
12623	*H*. *vespertilio*	Lectotype	Italy, Verona	[1780]	679.0
12624	*H*. *livornica*	Lectotype	Italy, Livorno	[1779]	749.0
12625	*H*. *livornica*	Paralectotype	Italy, Livorno	[1779]	73.5
12626	*H*. *livornica*	Paralectotype	Italy, Livorno	[1779]	263.2

All five specimens, including the three illustrated in Fig 4, exhibit no significant external change or post-extraction damage (see also S1 Fig), although one leg was detached and glued carefully back onto each specimen.

Minimal-destructive DNA extraction from Lepidoptera is challenging, as their body is often covered–at least partly—in a dense vestiture of scales [68] which fall off if physically manipulated [69]. However, they are an important trait to assist species identification by the variegated patterns they produce [47, 68, 70, 71]. Therefore, the detachment of scales has to be avoided in handling the specimens. There have already been approaches to none- or minimal destructive DNA extraction of museum insect specimens [2, 25, 47, 72], which mainly focus on the extraction of DNA from the whole specimen. These methods are not applicable to Lepidoptera, as this kind of handling would destroy most of the habitus of the specimen (including the detachment of wings and loss of most of the scales). Applicable methods for DNA extraction in Lepidoptera included the usage of the whole abdomen [49, 73, 74]. In those approaches, the habitus of the specimen was altered by the removal of the abdomen, the dissection of the genitalia and loss of scales at the abdomen while handling and during maceration for genitalia preparation. This kind of sampling is of great value if the genitalia must be examined, as the specimen will be damaged anyway, but should be avoided if the sample is of particular value.

The usage of small parts of the insects for minimal-destructive DNA extraction (e.g. destruction only of the body parts used) was already shown to be successful in Diptera, Hymenoptera and Lepidoptera, with the specimen retained in a museum as a voucher [14, 75–78]. Legs are particularly useful in Lepidoptera in contrast to bigger body parts (e.g. the abdomen or the whole body), since they dry fast and therefore do not provide a moist environment in which cellular endonucleases can continue their activity for a long time [14, 17]. However, legs provide in the Lepidoptera several traits which are used in research, from basic taxonomic identifications to morpho-functional studies and phylogenetic reconstructions (e.g. vestiture and colouration, spur formulae, biometric ratios, number and arrangement of spines, presence of scent tufts or arolia, sensilla ultrastructure), thus the destruction of these, especially in unique type specimens, leads to a loss of value for such analyses [69, 79]. Our study shows that using the Monarch kit with slight protocol adjustments, destruction of legs is no longer necessary so that the legs can be glued back to the specimen preserving every original morphological trait (Fig 4).

It is common practice with Lepidoptera type specimens to use one leg for DNA extraction without destruction of the leg [80–84]. The results of these approaches vary greatly, between 0.01 and 2.5 ng/μl [80, 81, 85, 86]. As only concentrations, but no extraction volumina or total DNA amounts are given in these studies, comparison of extracted DNA is only vaguely possible. The mean concentration of DNA in the type specimens used is 12.1 ng/μl (SD ± 6.7). Presuming an identical volume of 30 μl used in the cited studies [80], this equals to 9-fold more extracted DNA on average with our protocol compared to those published for Lepidoptera type specimens.

### Shotgun libraries

**Fig 5:**
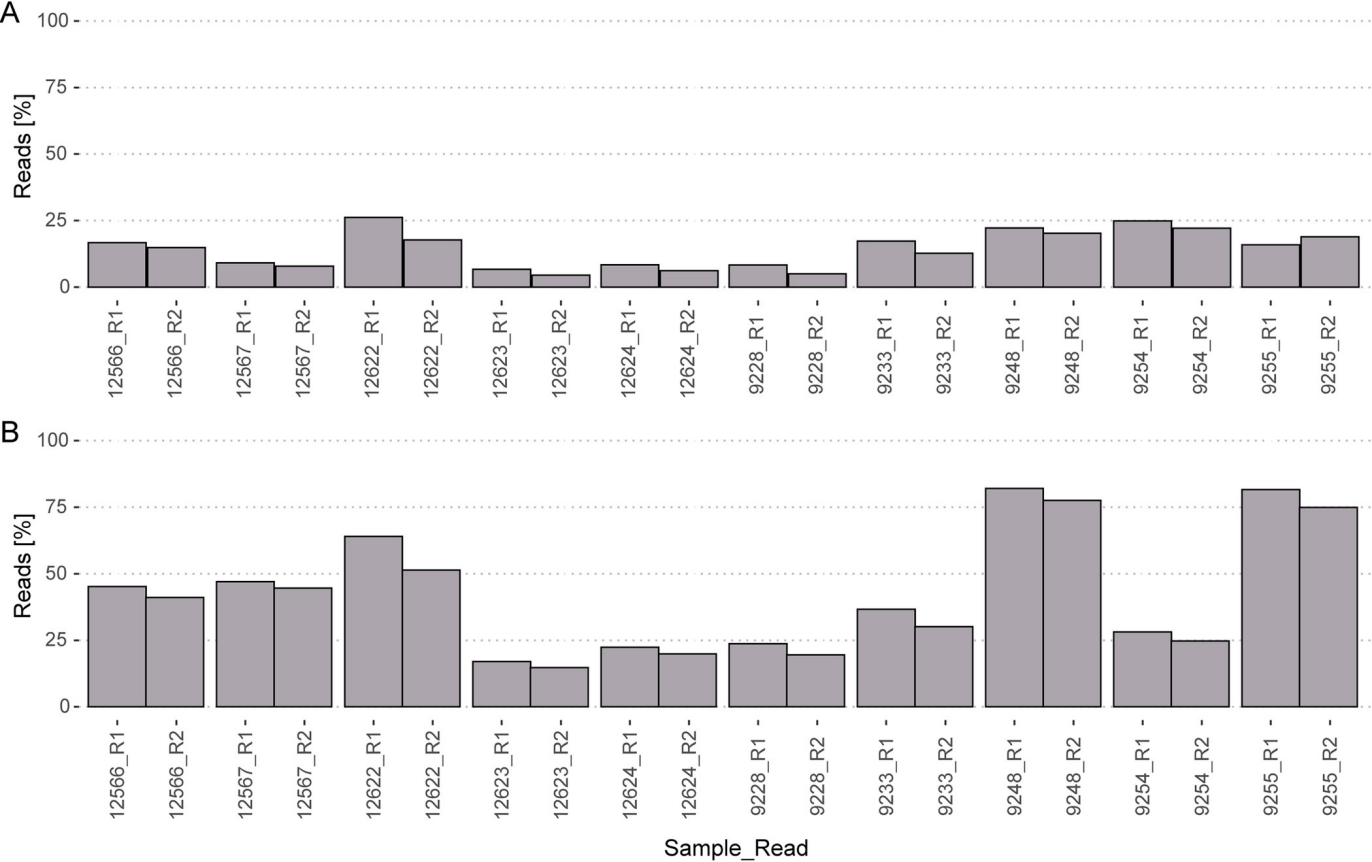
Raw read composition, showing the percentage of adapter content (A) and percentage of duplicates (B). Both Reads (R1 and R2) are shown for each sample.

We sequenced ten shotgun libraries on a 75 bp paired-end Illumina MiSeq run to test whether the Monarch extracted DNA is actually useful for NGS approaches. A total of 35.6 million demultiplexed raw reads resulting in an average of 1.78 million reads per individual were retained for further analysis (S3 Table). The raw reads of all samples exhibited in the mean 14.3% adapter contamination (SD ± 5.9%) and 42.3% duplicates (SD ± 18.8, Fig 5). The duplicate content is very variable due to different levels of DNA degradation between samples. These values were to be expected in highly degraded DNA samples and are comparable to other studies [25, 87, 88].

**Fig 6:**
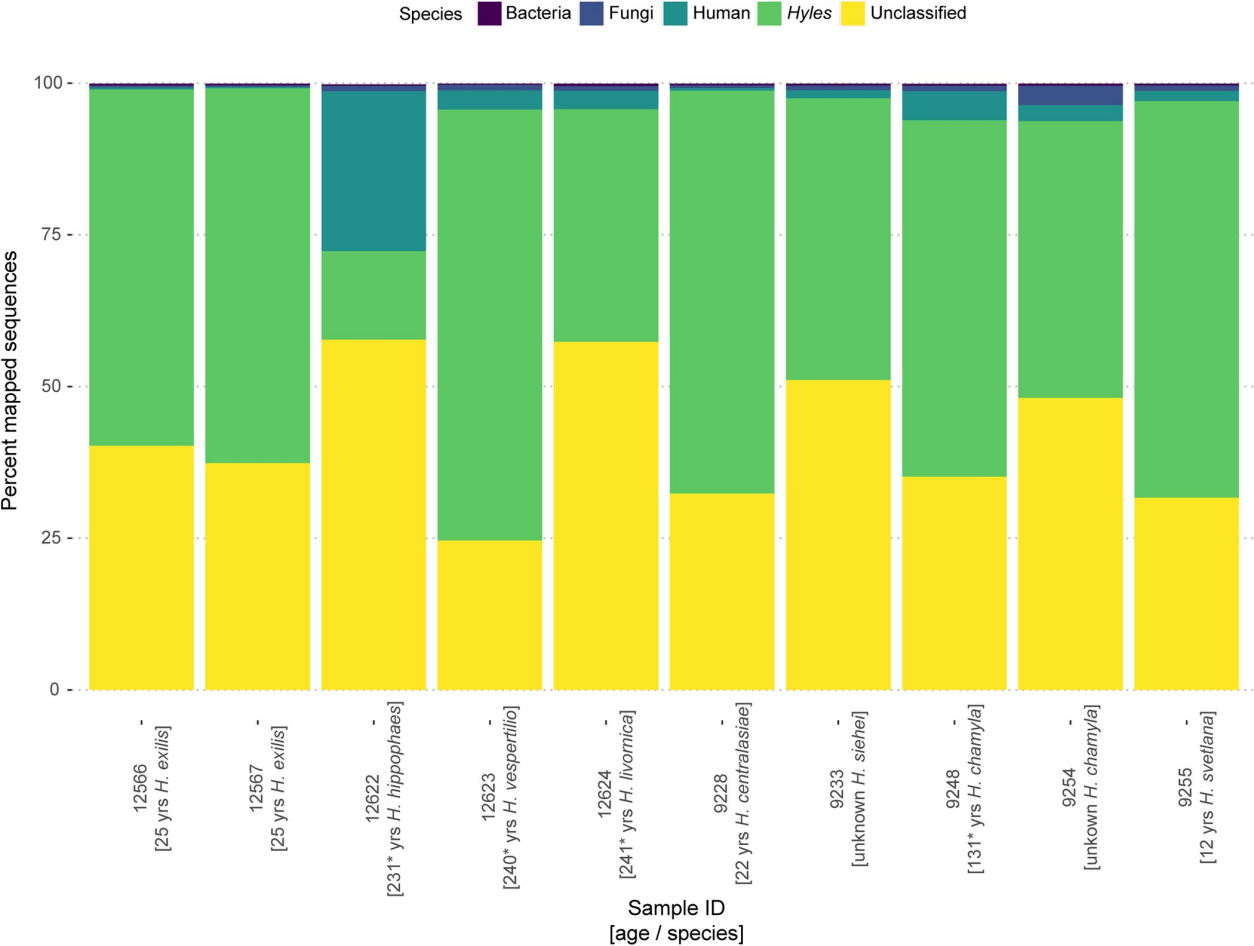
Percentage of endogenous DNA content of sequenced whole genome shotgun libraries. Sample age and species are added to the specimens MTD-TW number. Age is estimated in samples marked with * (see also S3 Table).

Overall, we were able to identify 52.7% (SD ± 13.2%) of endogenous DNA, mapping uniquely to the *Hyles* reference genome (Fig 6). The content of endogenous DNA is congruent with the findings in other studies where material from natural history collections was used [24, 25, 27], although we found a higher percentage of overall mapping success. Similar studies report mapping rates of about 40% [89, 90], while few reported mapping rates of > 50% [91] which is more similar to our results and closer to the mapping rates achieved using high-quality DNA [92].

The contamination content with DNA from bacteria, fungi and human was low with 1.9% (SD ± 2.1%) but varied between samples (Fig 6). One specimen, MTD-TW 12622, exhibited a higher percentage of contamination in relation to the endogenous DNA. In this sample human DNA was found in 8.8% of all sequences, while only 9.2% of the sequences were mapping to *Hyles* (see S3 Table). Exogenous DNA content in other whole genome shotgun sequencing studies of historical material varies greatly between the tissue tested and the possibility to clean the tissue before extraction (human DNA 0.1%– 9.8%, microbial DNA 0.01%– 45% [57, 58, 60–65]). But especially tissue such as skin and hair show similar levels of observed contamination, which cannot be decontaminated before DNA extraction [93–99]. This is also true for the chitinous exoskeleton of Lepidoptera, which is less resistant than bones and teeth to typical cleaning procedures, involving UV-light and chlorine [2]. The usage of these to remove contaminant DNA would lead to full degradation of the endogenous DNA inside of the legs and destruction of the scale structure. Hence such cleaning procedures are not suitable for legs from Lepidoptera specimens.

All samples showed a high percentage of unclassified sequences with a mean of 41.6% (SD ± 9.6%, Fig 6). This finding is consistent with the percentage of unmapped reads in other studies, where both different organisms and other DNA extraction methods were used [89, 90]. This was to be expected in whole genome shotgun sequences from museum samples because of the post-mortem DNA modifications [25, 90]. Overall the sequences obtained from the Monarch kit extracts are comparable with the quality of shotgun sequences from other museum material in the literature, while the age of our extracted specimens is exceptional among studies including dry pinned insect specimens [89, 100, 101]. Nevertheless, enrichment of these shotgun libraries will be necessary for higher sequencing efficiency [87–89, 98, 102, 103].

### Multi sequence alignment and phylogenetic tree

**Fig 7:**
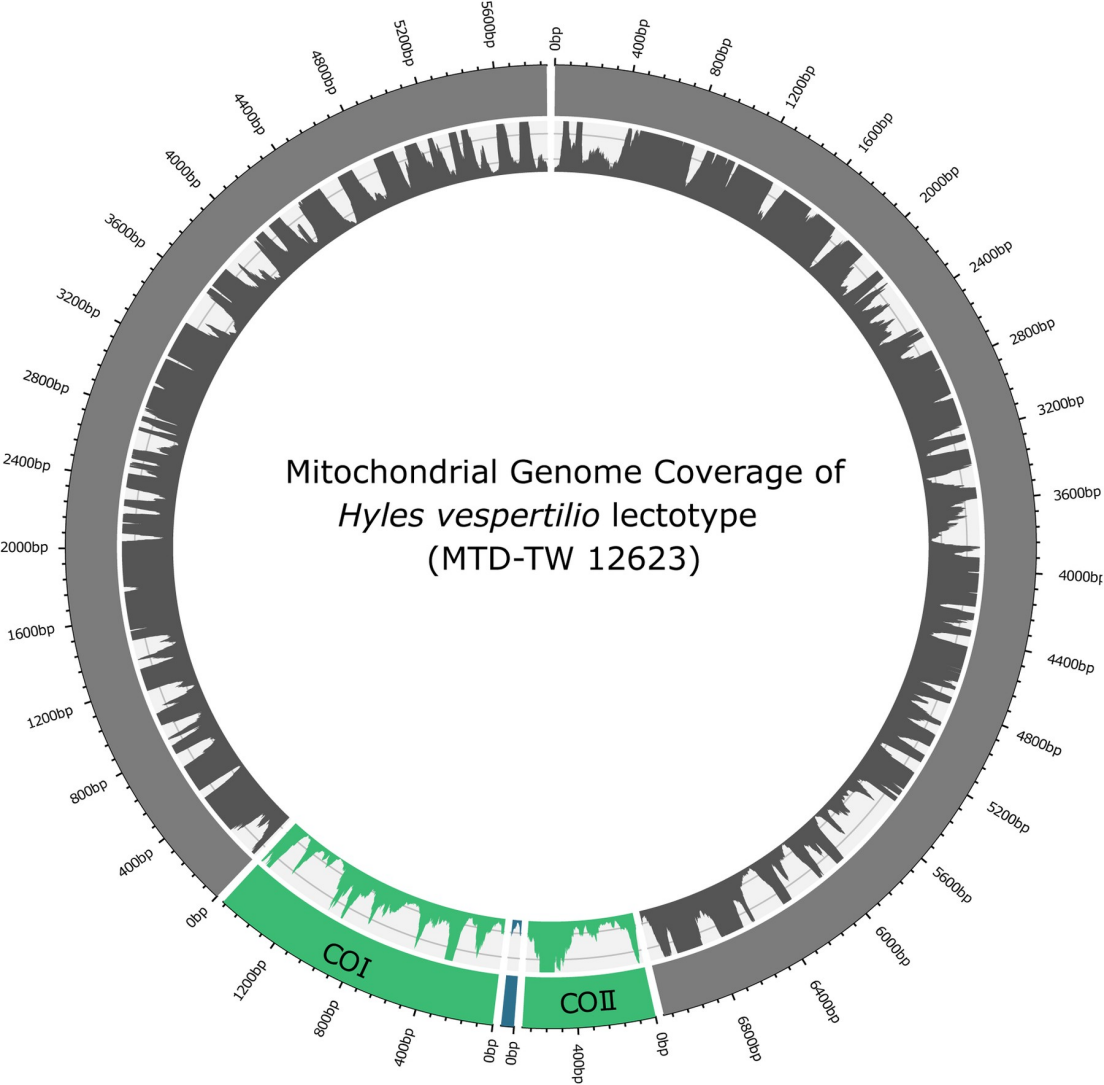
Coverage breadth and depth of the *Hyles vespertilio* lectotype (MTD-TW 12623) sequences along the mitochondrial reference genome of the same species (ZITAT). Coverage depth is scaled to a max depth of 100 to enable visibility of coverage depth <100X. The inner circle shows the coverage breadth and depth for this sample. The outer circle shows the mitochondrial reference genome with the annotated diagnostic subunits of the cytochrome oxidase subunits (COI and COII, green) and tRNA^Leu^ (trnL2, blue).

The mapping on the mitochondrial reference genome of *Hyles vespertilio* [32] resulted in a mean alignment length of 14,995 bp (SD ± 367 bp), equal to 98.0% (SD ± 2.4%) of the reference genome covered (Fig 7). The length of the alignment on the nuclear genome was in the mean 19,157,397 bp (SD ± 12,820,375 bp) long, covering 2.9% (SD ± 1.9%) of the nuclear reference genome (coverage for each individual can be found in S4 Table).

The comparison of the absolute alignment lengths with those in other studies focused on Lepidoptera is not meaningful, as the size of nuclear and mitochondrial genomes differ tremendously between different genera [104]. But the comparison of relative coverage of the alignment on the reference genome appears to be a useful measure to judge the quality of the sequenced reads.

In a recent study, the authors were able to cover 77.8% (SD ± 6.1) of the skipper butterfly mitochondrial reference genome [105], and 2.1% (SD ± 0.5%) of the nuclear reference genome [80, 106]. The relative coverage of the nuclear genome of both alignments is almost identical. In contrast, the relative coverage of the mitochondrial genome of our alignment is 20.2% higher than the one from the article. There could be multiple reasons for the difference in alignments lengths. First, there could be differences in data preparation, the used aligner and different settings used for the aligner, which can influence the amount of sequences mapped and therefore the length of the alignment [107]. As details on the data handling of the cited study are unknown to the authors, we could not test this hypothesis. Furthermore, the extraction method and concentrations found in this article (0.01–2.50 ng/μl) is very close to the concentrations observed with the Qiagen DNeasy Kit protocol, which we compared to the Monarch Oligo extraction protocol (see above). Therefore, it is possible that we extracted overall more DNA than the authors of the cited study by using the Monarch Oligo Clean-up protocol. While no values for volumes or the amount of extracted DNA are given, we can only add findings in other studies to this assumption, where it was shown that DNA extracted from museum samples has a representation bias to mitochondrial DNA [60]. Therefore, a higher total amount of extracted DNA would lead to more extracted mitochondrial than nuclear DNA.

To test the integrity of the extracted DNA from the Monarch Oligo extraction protocol, we calculated a phylogenetic tree using the diagnostic cytochrome oxidase subunits (COI and COII) and tRNA^Leu^ (trnL2) from the alignments performed with sequences from the same or related species of the genus *Hyles*, obtained from NCBI. To account for possible independence in the evolutionary models of the genes analysed, we calculated how many independent partitions could be defined in the alignment. By using PartitionFinder2 [40] we found two partitions (Table 4), with the first subset including mainly the first codon positions and the second codon positions of COII and tRNA^Leu^, while the second subset covered the third codon positions and the second codon position of COI.

**Table 4:** Best partition scheme from PartitionFinder2.

Subset	Best Model	# sites	Partition names
1	GTR+G+X	1044	coII_1st, coII_2nd, trnL2, co1_1st
2	GTR+I+G+X	1262	coII_3nd, co1_3nd, co1_2nd

These two partitions appeared to be appropriate from all we know about the different evolutionary rates and selection pressures of the different codon positions and protein-coding genes compared to non-protein-coding genes [108–111].

Therefore, the partitions and models in Table 4 were used in the phylogenetic tree reconstruction. While computing the phylogenetic tree, two of the 43 sequences failed the Chi^2^-test (FN386584 and MTD-TW 12622, p-value<5%; df = 3). MTD-TW 12622 failed most likely due to the high percentage of missing data (26.4%), which is the highest amount of missing data found in this alignment. Nevertheless, these two sequences were used for the tree reconstruction to provide the full picture of the sequences used here.

**Fig 8:**
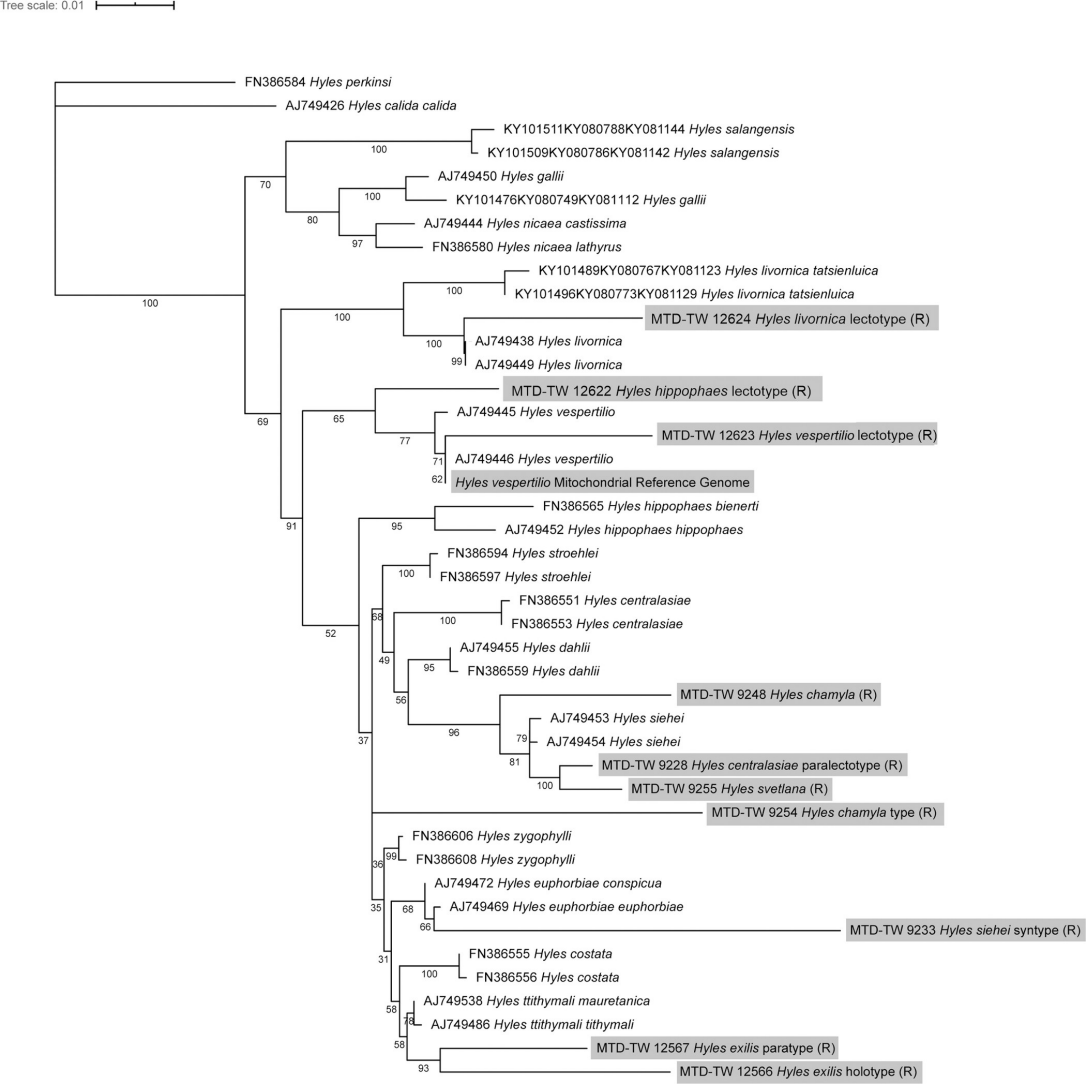
Phylogenetic tree based on the 2,306 bp multi sequence alignment of 43 sequences. The reconstruction is based on sequence data comprising the cytochrome oxidase subunits COI and COII and the intermediate tRNA^Leu^ (trnL2) gene fragments. If the reverse complementary sequences were used for the alignment, they were marked with (R).

In the phylogenetic tree (Fig 8) the samples MTD-TW 12624 (*H*. *livornica*), 12623 (*H*. *vespertilio*), 12566, 12567 (both *H*. *exilis*) and the *Hyles vespertilio* reference sequence grouped together with either the same or closely related species with moderate (71%), good (93%) or full bootstrap support. The samples MTD-TW 12622, 9248, 9288, 9255, 9254 and 9233 did not group as expected, while still showing high support values. But the more basal nodes show lower support values (bootstrap value <50%) which resembles the low support values found in the study from which the other sequences used originate (33).

This behaviour could be due to multiple reasons, starting with possible contamination during library preparation. By controlling for the positions on the 96-well plate where the libraries of the samples were prepared, no species in the neighbouring wells were found which could explain this pattern. Therefore, contamination during library preparation does not appear to be the reason for the detected pattern.

Moreover, this could be a real biological signal. The close relationship and species status of the Palaearctic *Hyles* species is strongly under debate, as hybridisation events between several of these taxa are well documented [112, 113]. This is especially the case for *Hyles siehei* and *Hyles centralasiae* hybridisation events [114]. The splitting of *H*. *centralasiae* into two branches separated by other species, including *H*. *siehei*, could suggest two mitochondrial lineages within this species, with the paralectotype (MTD-TW 9228) showing one mitochondrial lineage and the two samples FN386551 and FN386553 representing another lineage. The origin and species status of *Hyles svetlana* is not yet clear and is still discussed [115, 116], but the grouping in the cluster including some *H*. *siehei* and the *H*. *centralasiae* paralectotype could point to a hybrid origin of this entity. This would be in line with the original species description, in which *H*. *svetlana* was described as a subspecies of *H*. *siehei* based on mitochondrial DNA despite being closer phenotypically and in life history traits to *H*. *centralasiae* [115]. Only later it was raised to species status because of its biological differences to nominotypical *H*. *siehei* [114].

Furthermore the *H*. *siehei* syntype MTD-TW 9233 clusters together with *H*. *euphorbiae*, which raises the hypothesis that this individual is a hybrid of *H*. *siehei* with *H*. *euphorbiae* (subspecies). Indeed, this individual shows a transitional phenotype to *H*. *euphorbiae conspicua* (S2 Fig), which is at times found in *H*. *siehei* specimens and could validate its hybrid origin [114]. The polyphyletic pattern retrieved for the *H*. *centralasiae* / *H*. *siehei* / *H*. *svetlana* group raises several questions about their species status and possible hybridisation with other species. Answering these is beyond the scope of this work and will be examined more in depth later.

The position of the *Hyles hippophaes* lectotype (MTD-TW 12622) as sister to *Hyles vespertilio* could possibly be explained by the high percentage of missing data. Although reference alleles were masked out before we called the consensus sequence, it is still a possibility that the generation of the short read alignment using the *H*. *vespertilio* reference sequence influenced the alignment [116]. Anyway, a close relationship between *H*. *hippophaes* and *H*. *vespertilio* was already shown in other publications [113] and is recovered also in this analysis, as the two appear to be sister clades.

All samples used here show very long branches compared to the samples from earlier publications. Besides possible differences caused by the typical damage patterns introduced over time post-mortem [22], the samples used here are up to 241 years old, a time span accounting for multitude of generations separating these individuals from most of the more modern samples in the NCBI database (see also 33). This high number of generations separating the samples is the most likely reason explaining the high divergence over time between the individuals of the same species, equaling to longer branches in the tree [117].

### Conclusion

In this study, we reported and compared the performance of the Oligonucleotide Clean-up protocol of the Monarch® PCR & DNA Clean-up Kit for DNA extraction from museum specimens with those of the DNeasy extraction kit and the innuPREP DNA Mini Kit. We tested all three kits in a destructive approach where ground insect legs were used for DNA extraction to enable comparisons. In this approach, we were able to show that DNA extraction was successful in all tested specimens when using the Monarch® PCR & DNA Clean-up Kit. At the same time, the extractions of the other two kits started to fail in specimens which were 20 years and older. In the direct comparison of extracted DNA amounts from the same specimens, the Monarch® PCR & DNA Clean-up Kit yielded in the mean 17.3 times more DNA than the DNeasy extraction kit.

Additionally, we tested the application of the Monarch® PCR & DNA Clean-up Kit to a minimal-destructive extraction of DNA from type specimens. These specimens were up to 241 years old, and all yielded sufficient amounts of DNA suitable for sequencing projects. Therefore, we can conclude that the Oligonucleotide Clean-up protocol of the Monarch® PCR & DNA Clean-up Kit enabled us to:

- extract more DNA from older museum specimens in comparison to the other tested kits (destructive method),
- successfully extract DNA from century-old museum and type specimens while leaving the habitus of the leg used intact (minimal-destructive method).

The sequences obtained were suitable for downstream analyses, including phylogenetic tree reconstruction.

Although this approach should be tested in a broader spectrum of taxa for universal application, it appears to be useful for the DNA extraction from century-old museum and type specimens in Lepidoptera. By giving access to the information hidden in those specimens, it should enable researchers to answer an extensive range of evolutionary and ecological questions in the context of museum genomics.

## Supporting information

S1 Fig Two *Hyles* type specimens before and after sampling. Photographs of two moth specimens before (A, B) and after (a, b) the extraction, treatment and reattachment of one leg each (middle leg). The specimens individual MTD-TW numbers are: A/a 12622, B/b 12623 (Tab. S1).
(PDF)

S2 Fig Photo of MTD-TW 9233 *Hyles siehei* syntype, showing the transitional habitus to *H*. *euphorbiae conspicua*. (PDF)

S1 Table Comprehensive overview for all samples and extractions. (PDF)

S2 Table Extracted DNA amounts from six specimens of *Hyles chamyla* and *H*. *svetlana* comparing the DNeasy and the Monarch protocol. (PDF)

S3 Table Shotgun libraries of 10 specimens, identity of sequences were assigned using Kraken2. (PDF)

S4 Table Coverage summary for alignments of the shotgun libraries of 10 specimens. (PDF)
