# Supplementary material for: Advantages of an easy-to-use DNA extraction method for minimal-destructive analysis of collection specimens
Source: PLoS One. 2020 Jul 8;15(7):e0235222. doi: 10.1371/journal.pone.0235222 (PMC7343169; doi:10.1371/journal.pone.0235222)
Supplement: S1 Fig — Photographs of two moth specimens before (A, B) and after (a, b) the extraction, treatment and reattachment of one leg each (middle leg). The specimens individual MTD-TW numbers are: A/a 12622, B/b 12623 (Tab. S1). (PDF) [file pone.0235222.s001.pdf]

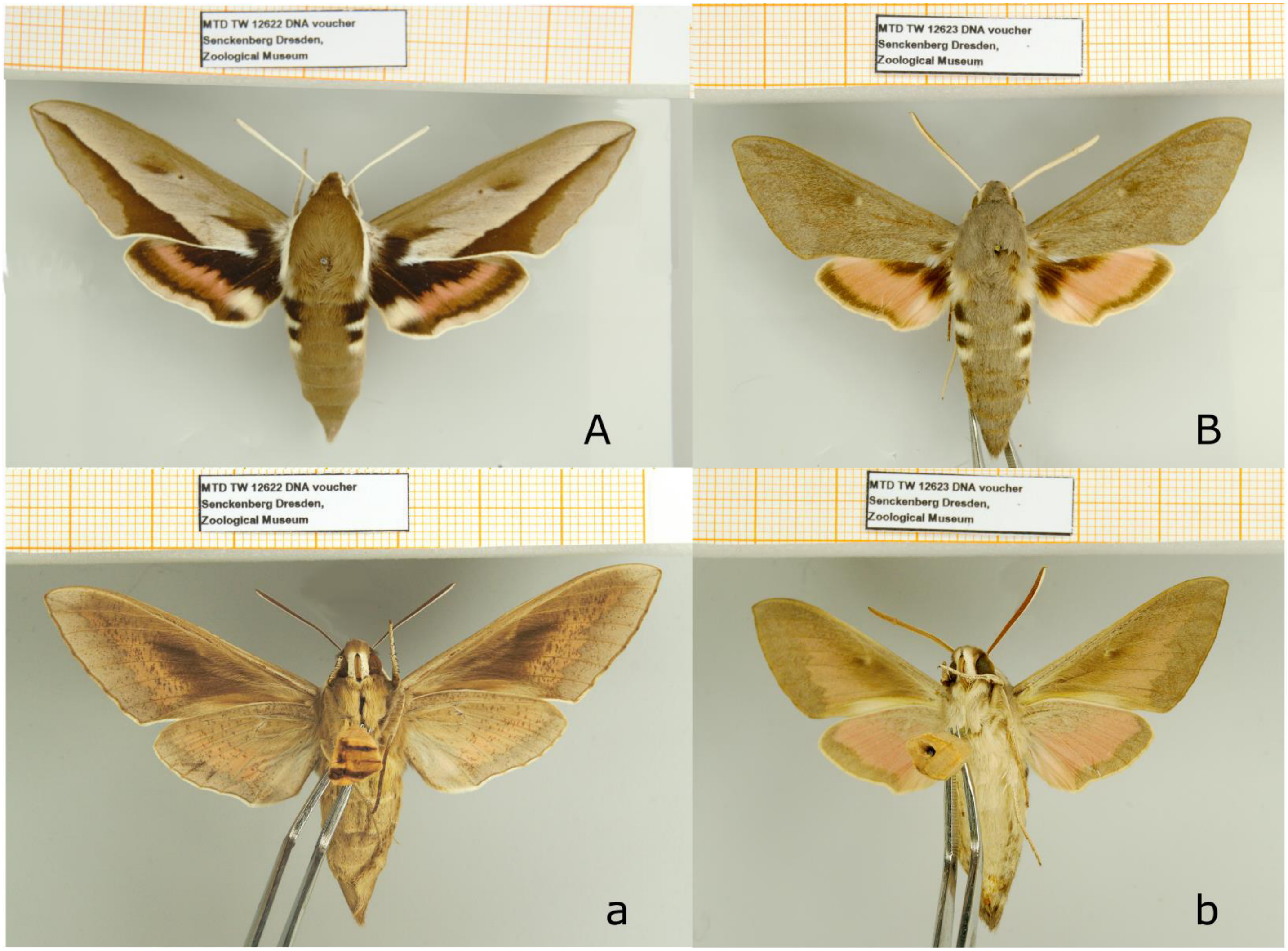

S3 Figure. Two *Hyles* type specimens before and after sampling. Photographs of two moth specimens before (A, B) and after (a, b) the extraction, treatment and reattachment of one leg each (middle leg). The specimens individual MTD-TW numbers are: A/a 12622, B/b 12623 (Tab. S1).
