# Supplementary material for: Advantages of an easy-to-use DNA extraction method for minimal-destructive analysis of collection specimens
Source: PLoS One. 2020 Jul 8;15(7):e0235222. doi: 10.1371/journal.pone.0235222 (PMC7343169; doi:10.1371/journal.pone.0235222)
Supplement: S2 Fig — (PDF) [file pone.0235222.s002.pdf]

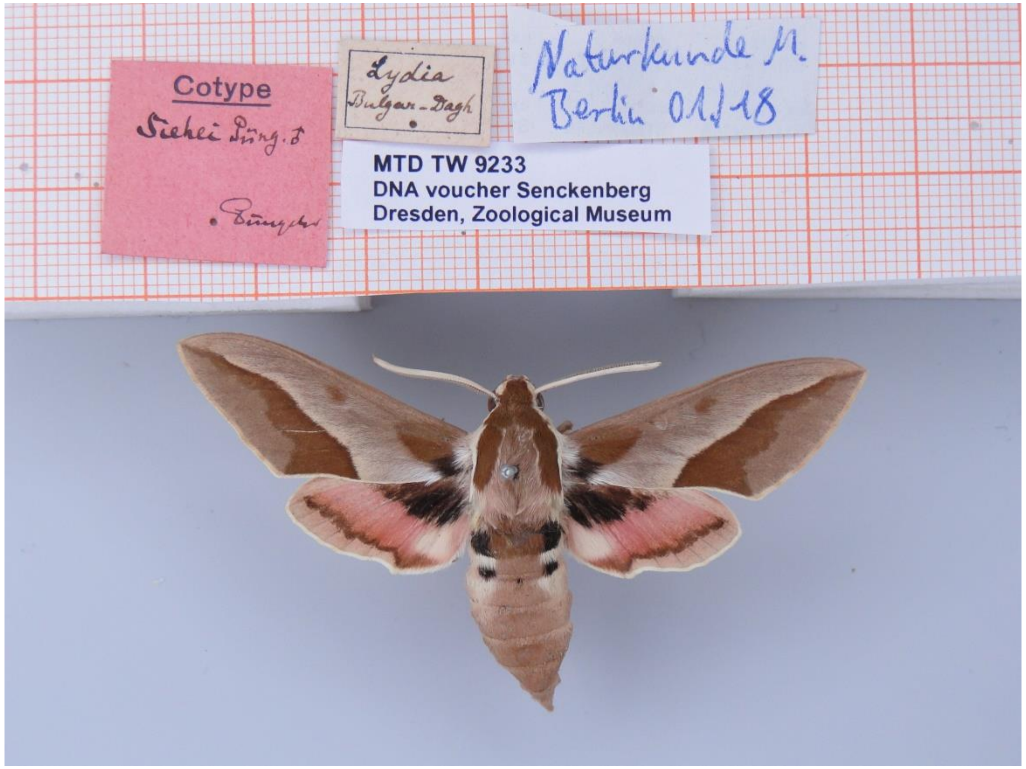

S6 Figure. Photo of MTD-TW 9233 *Hyles siehei* syntype, showing the translational habitus to *Hyles euphorbiae conspicua*.
