# Supplementary material for: Advantages of an easy-to-use DNA extraction method for minimal-destructive analysis of collection specimens
Source: PLoS One. 2020 Jul 8;15(7):e0235222. doi: 10.1371/journal.pone.0235222 (PMC7343169; doi:10.1371/journal.pone.0235222)
Supplement: S1 Table — (PDF) [file pone.0235222.s003.pdf]

S1 Table Comprehensive overview for all samples and extractions.

ID	MTD-TW	Species	Location	Collector	Museum	Collection Year	Extraction protocol	Total DNA (ng)
HLi_1066	1066	*Hyles livornica*	Israel	leg. Müller	Bavarian State Collection of Zoology, Munich, Germany	10.02.1999	Monarch PCR&DNA Clean up Kit	131.60
HCo_1321	1321	*Hyles costata*	Mongolia	Tz. Odbayar	Senckenberg Natural History Collections, Dresden, Germany	14.06.2005	Monarch PCR&DNA Clean up Kit	357.00
HNe_1717	1717	*Hyles nervosa*	Pakistan	Ron Brechlin	Collection Ronald Brechlin, Pasewalk, Germany	19.05.1999	Monarch PCR&DNA Clean up Kit	65.45
HNe_1720	1720	*Hyles nervosa*	Pakistan	Csovari & Mikus	Collection Ronald Brechlin, Pasewalk, Germany	30.07.1998	Monarch PCR&DNA Clean up Kit	63.70
HSt_1721	1721	*Hyles stroehlei*	Pakistan	Ron Brechlin	Collection Ronald Brechlin, Pasewalk, Germany	11.08.1998	Monarch PCR&DNA Clean up Kit	112.70
HSt_1722	1722	*Hyles stroehlei*	Pakistan	Ron Brechlin	Collection Ronald Brechlin, Pasewalk, Germany	18.07.1999	Monarch PCR&DNA Clean up Kit	65.80
HRo_1723	1723	*Hyles robertsi elisabethae*	Pakistan	Ron Brechlin	Collection Ronald Brechlin, Pasewalk, Germany	18.04.1999	DNeasy Blood and tissue	0.00
HCh_1726	1726	*Hyles chamyla*	Mongolia	Saldaitis	Collection Ronald Brechlin, Pasewalk, Germany	06.06.2004	innuPREP DNA MIni Kit	59.60
HAp_1728	1728	*Hyles apocyni*	Tadjikistan	Dr. Ronald Brechlin	Collection Ronald Brechlin, Pasewalk, Germany	10.08.2000	innuPREP DNA MIni Kit	308.00
HNi_3081	3081	*Hyles nicaea*	Iran	leg. Schurian & ten Hagen	Collection Schurian & ten Hagen	04.07.2000	Monarch PCR&DNA Clean up Kit	155.40
HLi_3462	3462	*Hyles livornica*	Kasachstan	Trofimova T.A. and Shovkoon D.F.	Samara University, Faculty of Biology, Russia	25.05.2006	Monarch PCR&DNA Clean up Kit	137.90
HGa_3463	3463	*Hyles gallii*	Russia	D. Shovkun	Samara University, Faculty of Biology, Russia	30.06.2007	Monarch PCR&DNA Clean up Kit	164.15
HGa_3464	3464	*Hyles gallii*	Kasachstan	Trofimova T.A. and Shovkoon D.F.	Samara University, Faculty of Biology, Russia	15.05.2006	Monarch PCR&DNA Clean up Kit	241.50
HCo_3512	3512	*Hyles costata*	Mongolia	Dr. Z. Kaszab	Hungarian Natural History Museum	02.08.1965	Monarch PCR&DNA Clean up Kit	348.60
HVe_4402	4402	*Hyles vespertilio*	Russland	Gary Saunders	Senckenberg Natural History Collections, Dresden, Germany	2008	Monarch PCR&DNA Clean up Kit	196.35
HSi_8965	8965	*Hyles siehei*	Türkei	H.Harbich	Senckenberg Natural History Collections, Dresden, Germany	1998	innuPREP DNA MIni Kit	0.00
HCe_9034	9034	*Hyles centralasiae complex*	Kasachstan	M.Danilevski,	Entomological Museum, Marktleuthen, Germany	07.05.2001	innuPREP DNA MIni Kit	117.00
HCe_9036	9036	*Hyles centralasiae complex*	Kasachstan	M.Danilevski	Entomological Museum, Marktleuthen, Germany	24.04.2000	DNeasy Blood and tissue	35.56
HCe_9041PT	9041	*Hyles centralasiae complex*	Kasachstan	TrofimovaTA&ShovkoonDF	Entomological Museum, Marktleuthen, Germany	15.05.2006	DNeasy Blood and tissue	48.72
HCe_9042	9042	*Hyles centralasiae complex*	Kasachstan	M.Danilevski	Entomological Museum, Marktleuthen, Germany	29.04.2000	DNeasy Blood and tissue	56.28
HCe_9051	9051	*Hyles centralasiae complex*	Türkei	deFreina	Museum Witt, München, Germany	8.-9.6.2002	DNeasy Blood and tissue	54.88
HCo_9121	9121	*Hyles costata / exilis*	Russia	R.V. Yakovlev	Collection Roman Yakovlev, Barnaul, Russia	29.05.2017	Monarch PCR&DNA Clean up Kit	289.80
HGa_9123	9123	*Hyles gallii*	Kasachstan	V. Zurilina	Collection Roman Yakovlev, Barnaul, Russia	18.07.2009	Monarch PCR&DNA Clean up Kit	50.05
HGa_9124	9124	*Hyles gallii*	Kasachstan	R.V. Yakovlev	Collection Roman Yakovlev, Barnaul, Russia	11-13.06.2012	Monarch PCR&DNA Clean up Kit	113.05
HGa_9127	9127	*Hyles gallii*	Russia	R. Yakovlev, Yu. Perunov & P. Ustjuzhanin	Collection Roman Yakovlev, Barnaul, Russia	20−21.07.2012	Monarch PCR&DNA Clean up Kit	169.05
HGa_9128	9128	*Hyles gallii*	Russia	V. Zurilina	Collection Roman Yakovlev, Barnaul, Russia	12.07.2011	Monarch PCR&DNA Clean up Kit	79.45
HHi_9129	9129	*Hyles hippophaes*	Kazakhstan	S. Rybalkin & R. Yakovlev	Collection Roman Yakovlev, Barnaul, Russia	01.07.2012	DNeasy Blood and tissue	26.18
HLi_9130	9130	*Hyles livornica*	Mongolia	R. Yakovlev	Collection Roman Yakovlev, Barnaul, Russia	3.-4.06.2011	Monarch PCR&DNA Clean up Kit	350.00
HHi_9131	9131	*Hyles hippophaes*	Russia	R. Yakovlev, Yu. Perunov & P. Ustjuzhanin	Collection Roman Yakovlev, Barnaul, Russia	20−21.07.2012	DNeasy Blood and tissue	8.12
HCh_9144	9144	*Hyles chamyla oder apocyni*	Mongolia	R.V.Yakovlev	Collection Roman Yakovlev, Barnaul, Russia	26−28.06.2017	innuPREP DNA MIni Kit	81.80
HSp_9147	9147	*Hyles sp.*	Russia	R.V.Yakovlev	Collection Roman Yakovlev, Barnaul, Russia	02−03.06.2017	innuPREP DNA MIni Kit	510.00
HCh_9152	9152	*Hyles chamyla*	Mongolia	R.V.Yakovlev	Collection Roman Yakovlev, Barnaul, Russia	26−28.06.2017	innuPREP DNA MIni Kit	660.00
HHi_9155	9155	*Hyles hippophaes*	Kasachstan	S.Rybalkin&R.Yakovlev	Collection Roman Yakovlev, Barnaul, Russia	01.07.2012	DNeasy Blood and tissue	129.50
HSp_9167	9167	*Hyles sp.*	Mongolia	R.V.Yakovlev	Collection Roman Yakovlev, Barnaul, Russia	28.06.2017	innuPREP DNA MIni Kit	236.00
HSp_9171	9171	*Hyles sp.*	Mongolia	R.V.Yakovlev	Collection Roman Yakovlev, Barnaul, Russia	26−28.06.2017	innuPREP DNA MIni Kit	266.00
HSp_9172	9172	*Hyles sp.*	Mongolia	R.V.Yakovlev	Collection Roman Yakovlev, Barnaul, Russia	26−28.06.2017	innuPREP DNA MIni Kit	108.00
HSv_9183	9183	*Hyles svetlana*	Kasachstan	P.Gorbunov	Collection Roman Yakovlev, Barnaul, Russia	24.05.2015	innuPREP DNA MIni Kit	60.20
HCh_9186	9186	*Hyles chamyla*	Mongolia	R.Yakovlev,E.Guskova	Collection Roman Yakovlev, Barnaul, Russia	9-10.07.2010	innuPREP DNA MIni Kit	308.00
HCh_9187	9187	*Hyles chamyla*	Mongolia	R.Yakovlev	Collection Roman Yakovlev, Barnaul, Russia	1-2.06.2011	DNeasy Blood and tissue	270.20
HHi_9190	9190	*Hyles hippophaeas*	Mongolia	R.Yakovlev	Collection Roman Yakovlev, Barnaul, Russia	07−08.07.2015	DNeasy Blood and tissue	396.20
HSv_9191	9191	*Hyles svetlana*	Kasachstan	P.Gorbunov	Collection Roman Yakovlev, Barnaul, Russia	24.05.2015	innuPREP DNA MIni Kit	12.00
HSv_9201	9201	*Hyles svetlana*	Mongolia	R.&A.Yakovlev	Collection Roman Yakovlev, Barnaul, Russia	18−21.05.2015	innuPREP DNA MIni Kit	61.00
HSv_9202	9202	*Hyles svetlana*	Kasachstan	P.Gorbunov	Collection Roman Yakovlev, Barnaul, Russia	24.05.2015	innuPREP DNA MIni Kit	42.60
HHi_9214	9214	*Hyles hippophaeas*	Mongolia	R.Yakovlev	Collection Roman Yakovlev, Barnaul, Russia	26.06.2015	DNeasy Blood and tissue	85.40
HCe_9227LT	9227	*Hyles centralasiae*	Usbekistan	ex coll. Staudinger	Museum of Natural History Berlin, Germany	1996	Monarch PCR&DNA Clean up Kit	211.75
HCe_9228PLT	9228	*Hyles centralasiae*	Usbekistan	ex coll. Staudinger	Museum of Natural History Berlin, Germany	1996	Monarch PCR&DNA Clean up Kit	159.25
HHi_9249LT	9249	*Hyles hippophaes bienerti*	Iran?	Eitschberger?	Museum of Natural History Berlin, Germany	1996	Monarch PCR&DNA Clean up Kit	79.45
HHi_9250PLT	9250	*Hyles hippophaes bienerti*	Iran?	Eitschberger?	Museum of Natural History Berlin, Germany	1996	Monarch PCR&DNA Clean up Kit	248.15
HCh_9252	9252	*Hyles chamyla*	Turkmenistan	Püngeler?	Museum of Natural History Berlin, Germany	1908	Monarch PCR&DNA Clean up Kit	353.50
HCh_9253	9253	*Hyles chamyla*	Türkei	Rückbeil?	Museum of Natural History Berlin, Germany	1908	Monarch PCR&DNA Clean up Kit	451.50
HCh_9254	9254	*Hyles chamyla*	China	Denso?	Museum of Natural History Berlin, Germany	1913	Monarch PCR&DNA Clean up Kit	413.00
HSv_9255PT	9255	*Hyles siehei svetlana*	Kasachstan	Shovkoon	Museum of Natural History Berlin, Germany	2006	Monarch PCR&DNA Clean up Kit	703.50
HSi_9267	9267	*Hyles siehei?*	Pakistan	-	Collection Rafaqat Masroor, Pakistan	2013	DNeasy Blood and tissue	348.60
HSi_9271	9271	*Hyles siehei?*	Pakistan	Fiaz	Collection Rafaqat Masroor, Pakistan	1990	DNeasy Blood and tissue	11.76
HHi_9278PT	9278	*Hyles hippophaes bienerti*	Kasachstan	V. Karalius & I. Miatleuski	Sphingidae museum s.r.o., Příbram, Czech republic	1999	Monarch PCR&DNA Clean up Kit	178.85
HHi_9279PT	9279	*Hyles hippophaes bienerti*	Kasachstan	V. Karalius & I. Miatleuski	Sphingidae museum s.r.o., Příbram, Czech republic	1999	Monarch PCR&DNA Clean up Kit	174.65
HHi_9287	9287	*Hyles hippophaes bienerti*	Pakistan	B.Benedek&J.Babies	Sphingidae museum s.r.o., Příbram, Czech republic	2014	DNeasy Blood and tissue	81.90
HHi_9288	9288	*Hyles hippophaes bienerti*	Pakistan	B.Benedek & J. Babies	Sphingidae museum s.r.o., Příbram, Czech republic	2014	DNeasy Blood and tissue	20.44
HHi_9289	9289	*Hyles hippophaes bienerti*	Pakistan	B.Benedek&J.Babies	Sphingidae museum s.r.o., Příbram, Czech republic	2014	DNeasy Blood and tissue	215.60
HHi_9290	9290	*Hyles hippophaes bienerti*	Tadjikistan	V.Gurko	Sphingidae museum s.r.o., Příbram, Czech republic	2014	DNeasy Blood and tissue	219.80
HHi_9291	9291	*Hyles hippophaes bienerti*	Tadjikistan	V.Gurko	Sphingidae museum s.r.o., Příbram, Czech republic	2002	DNeasy Blood and tissue	294.00
HRo_9296	9296	*Hyles robertsi*	Iran	Sphingidae museum, C.R.	Sphingidae museum s.r.o., Příbram, Czech republic	2010	DNeasy Blood and tissue	1820.00

S1 Table Comprehensive overview for all samples and extractions.

HAp_9297	9297	*Hyles apocyni*	Iran	Thomas Melichar and Halada	Sphingidae museum s.r.o., Příbram, Czech republic	2010	innuPREP DNA MIni Kit	1010.00
HAp_9298	9298	*Hyles apocyni*	Tadjikistan	A.Bergmann	Sphingidae museum s.r.o., Příbram, Czech republic	1999	innuPREP DNA MIni Kit	430.00
HAp_9299	9299	*Hyles apocyni*	Tadjikistan	A.Bergmann	Sphingidae museum s.r.o., Příbram, Czech republic	1999	innuPREP DNA MIni Kit	448.00
HAp_9300	9300	*Hyles apocyni*	Tadjikistan	A.Bergmann	Sphingidae museum s.r.o., Příbram, Czech republic	2000	innuPREP DNA MIni Kit	572.00
HSv_9311	9311	*les siehei svetlana/ Hyles centralas*	Kasachstan	Melichar	Sphingidae museum s.r.o., Příbram, Czech republic	2001	DNeasy Blood and tissue	32.48
HSv_9312	9312	*Hyles siehei svetlana*	Kasachstan	-	Sphingidae museum s.r.o., Příbram, Czech republic	1998	DNeasy Blood and tissue	14.42
HSv_9313	9313	*les siehei svetlana/ Hyles centralas*	Kasachstan	Melichar	Sphingidae museum s.r.o., Příbram, Czech republic	2001	DNeasy Blood and tissue	29.54
HSv_9314	9314	*les siehei svetlana/ Hyles centralas*	Kasachstan	Melichar	Sphingidae museum s.r.o., Příbram, Czech republic	2001	DNeasy Blood and tissue	16.10
HSi_9315	9315	*Hyles siehei*	Turkey	-	Sphingidae museum s.r.o., Příbram, Czech republic	2002	DNeasy Blood and tissue	247.80
HSi_9316	9316	*Hyles siehei*	Turkey	M.Geck	Sphingidae museum s.r.o., Příbram, Czech republic	2011	DNeasy Blood and tissue	847.00
HCH_9318	9318	*Hyles chamyla*	China	Dr. F. Karrer, CH-4800	Sphingidae museum s.r.o., Příbram, Czech republic	2002	DNeasy Blood and tissue	25.76
HCh_9319	9319	*Hyles chamyla*	China	Dr. F. Karrer, CH-4800	Sphingidae museum s.r.o., Příbram, Czech republic	2002	DNeasy Blood and tissue	32.48
HCh_9320	9320	*Hyles chamyla*	China	Dr.F.Karrer,CH-4802	Sphingidae museum s.r.o., Příbram, Czech republic	2002	innuPREP DNA MIni Kit	358.00
HRo_9321	9321	*Hyles robertsi*	Iran	-	Sphingidae museum s.r.o., Příbram, Czech republic	1999	DNeasy Blood and tissue	30.24
HRo_9322	9322	*Hyles robertsi*	Iran	Sphingidae museum C.R.	Sphingidae museum s.r.o., Příbram, Czech republic	2009	DNeasy Blood and tissue	26.04
HHi_11552	11552	*Hyles hippophaes*	Türkei	Kautt	Entomological Museum, Marktleuthen, Germany	1990	DNeasy Blood and tissue	16.10
HHi_11553	11553	*Hyles hippophaes*	Türkei	Groß	Entomological Museum, Marktleuthen, Germany	1979	DNeasy Blood and tissue	14.70
HHi_11554	11554	*Hyles hippophaes*	Armenien	Eitschberger EMEM	Entomological Museum, Marktleuthen, Germany	1997	DNeasy Blood and tissue	34.16
HHi_11555	11555	*Hyles hippophaes*	Turkmenistan	V.Perepel	Entomological Museum, Marktleuthen, Germany	1996	DNeasy Blood and tissue	32.34
HHi_11557	11557	*Hyles hippophaes*	Iran	Gy.Fábián,L.Szécsényi&K.Székely	Entomological Museum, Marktleuthen, Germany	2000	DNeasy Blood and tissue	15.54
HAp_11578	11578	*Hyles apocyni*	Tajikistan	V.&A.Lukhtanov	Entomological Museum, Marktleuthen, Germany	1994	DNeasy Blood and tissue	40.60
HAp_11580	11580	*Hyles apocyni*	Tajikistan	Lukhtanov	Entomological Museum, Marktleuthen, Germany	1994	DNeasy Blood and tissue	9.38
HAp_11581	11581	*Hyles apocyni*	Tajikistan	Lukhtanov	Entomological Museum, Marktleuthen, Germany	1994	DNeasy Blood and tissue	12.04
HCe_11589	11589	*Hyles centralasiae*	Usbekistan	V. & A. Lukhtanov	Entomological Museum, Marktleuthen, Germany	1994	DNeasy Blood and tissue	37.10
HCe_11590	11590	*Hyles centralasiae*	Tajikistan	V. Lukhtanov	Entomological Museum, Marktleuthen, Germany	1994	DNeasy Blood and tissue	34.44
HCe_11592	11592	*Hyles centralasiae*	Usbekistan	leg. Zolotuin	Entomological Museum, Marktleuthen, Germany	1992	DNeasy Blood and tissue	53.90
HCe_11593	11593	*Hyles centralasiae*	Usbekistan	V. Lukhtanov	Entomological Museum, Marktleuthen, Germany	1966	DNeasy Blood and tissue	572.60
HCe_11594	11594	*Hyles centralasiae*	Kirgisien	V.Lukhtanov	Entomological Museum, Marktleuthen, Germany	1996	DNeasy Blood and tissue	34.30
HCe_11595	11595	*Hyles centralasiae*	Kirgisien	V.Lukhtanov	Entomological Museum, Marktleuthen, Germany	1997	DNeasy Blood and tissue	32.76
HCe_11596	11596	*Hyles centralasiae*	Kirgisien	V.Lukhtanov	Entomological Museum, Marktleuthen, Germany	1996	DNeasy Blood and tissue	24.36
HZy_11599	11599	*Hyles zygophylli*	China	Floriani,Saldaitis	Entomological Museum, Marktleuthen, Germany	2008	DNeasy Blood and tissue	34.86
HRo_11600	11600	*Hyles robertsi*	Iran	A. Hofmann, J.-U. Meineke	Entomological Museum, Marktleuthen, Germany	1998	DNeasy Blood and tissue	784.00
HRo_11601	11601	*Hyles robertsi*	Iran	A.Hofmann,P.Kautt	Entomological Museum, Marktleuthen, Germany	1997	DNeasy Blood and tissue	48.16
HRo_11606	11606	*Hyles robertsi*	Turkmenistan	J.Miatleuski,A.Povilaitis	Entomological Museum, Marktleuthen, Germany	1996	innuPREP DNA MIni Kit	35.00
HRo_11609	11609	*Hyles robertsi*	Turkmenistan	Matlenski	Entomological Museum, Marktleuthen, Germany	1995	innuPREP DNA MIni Kit	41.00
HRo_11613	11613	*Hyles robertsi*	Turkmenistan	Matlenski	Entomological Museum, Marktleuthen, Germany	1996	innuPREP DNA MIni Kit	108.00
HRo_11614	11614	*Hyles robertsi*	Turkmenistan	Matlenski	Entomological Museum, Marktleuthen, Germany	1995	innuPREP DNA MIni Kit	124.00
HRo_11617	11617	*Hyles robertsi*	Turkmenistan	Matlenski	Entomological Museum, Marktleuthen, Germany	1995	innuPREP DNA MIni Kit	49.80
HEu_11630	11630	*Hyles euphorbiae*	Turkey	leg. Groß	Entomological Museum, Marktleuthen, Germany	1974	DNeasy Blood and tissue	331.80
HEu_11632	11632	*Hyles euphorbiae*	Armenia	A.Dantchenko	Entomological Museum, Marktleuthen, Germany	2001	DNeasy Blood and tissue	38.36
HEu_11642	11642	*Hyles euphorbiae*	Tajikisitan	-	Entomological Museum, Marktleuthen, Germany	2003	DNeasy Blood and tissue	37.24
HEu_11654	11654	*Hyles euphorbiae*	Kirgisien	S.Churkin	Entomological Museum, Marktleuthen, Germany	2000	DNeasy Blood and tissue	29.12
HEu_11655	11655	*Hyles euphorbiae*	Kirgisien	coll.A.Saldaitis	Entomological Museum, Marktleuthen, Germany	2000	DNeasy Blood and tissue	14.42
HEu_11658	11658	*Hyles euphorbiae*	Kasachstan	S.K.Korb	Entomological Museum, Marktleuthen, Germany	2010	DNeasy Blood and tissue	47.74
HCe_11664	11664	*Hyles centalasiae*	Kasachstan	V.Lukhtanov	Entomological Museum, Marktleuthen, Germany	1997	innuPREP DNA MIni Kit	49.40
HEu_11665	11665	*Hyles euphorbiae*	Kasachstan	KaraliusV.andMiatleuskiJ.	Entomological Museum, Marktleuthen, Germany	2000	DNeasy Blood and tissue	38.08
HZy_11690	11690	*Hyles zygophylli*	Kasachstan	V.Lukhtanov	Entomological Museum, Marktleuthen, Germany	2000	DNeasy Blood and tissue	0.00
HZy_11691	11691	*Hyles zygophylli*	Kasachstan	S.K.Korb	Entomological Museum, Marktleuthen, Germany	2011	DNeasy Blood and tissue	39.34
HZy_11692	11692	*Hyles zygophylli*	Kasachstan	S.K.Korb	Entomological Museum, Marktleuthen, Germany	2011	DNeasy Blood and tissue	58.10
HSi_11757	11757	*Hyles siehei*	Turkey	Geck	Entomological Collection II, Senckenberg, Frankfurt a.M., Germany	2000	innuPREP DNA MIni Kit	278.00
HCo_11850	11850	*Hyles costata/exilis/churkini*	Mongolia	Saldaites	Entomological Museum, Marktleuthen, Germany	2004	Monarch PCR&DNA Clean up Kit	186.90
HCo_11851	11851	*Hyles costata/exilis/churkini*	Siberia	Kruger, Saldaitis	Entomological Museum, Marktleuthen, Germany	2000	Monarch PCR&DNA Clean up Kit	216.30
HCo_11852	11852	*Hyles costata/exilis/churkini*	Siberia	Kruger, Saldaitis	Entomological Museum, Marktleuthen, Germany	2000	Monarch PCR&DNA Clean up Kit	207.90
HEu_11863	11863	*Hyles euphorbiae*	Kasachstan	leg. V.&A.Lukhtanov	Entomological Museum, Marktleuthen, Germany	1992	DNeasy Blood and tissue	714.00
HCo_11865	11865	*Hyles costata/exilis/churkini*	Kasachstan	V. Lukhtanov	Entomological Museum, Marktleuthen, Germany	1996	Monarch PCR&DNA Clean up Kit	318.50
HEu_11917	11917	*Hyles euphorbiae complex*	Algerien	Cult. F.Danner	Entomological Museum, Marktleuthen, Germany	05.-06.07.1997	DNeasy Blood and tissue	7.42
HEu_11919	11919	*Hyles euphorbiae complex*	Algerien	F. Danner cult.	Entomological Museum, Marktleuthen, Germany	10.09.1997	DNeasy Blood and tissue	10.64
HAp_11941	11941	*Hyles apocyni*	China	Floriani	Entomological Museum, Marktleuthen, Germany	2013	DNeasy Blood and tissue	9.80
HZy_11942	11942	*Hyles zygophylli*	Armenia	A.Dantchenko	Entomological Museum, Marktleuthen, Germany	2014	DNeasy Blood and tissue	46.62
HZy_11943	11943	*Hyles zygophylli*	Armenia	A.Dantchenko	Entomological Museum, Marktleuthen, Germany	1996	DNeasy Blood and tissue	16.38
HZy_11944	11944	*Hyles zygophylli*	Russland	Eitschberger EMEM	Entomological Museum, Marktleuthen, Germany	1989	DNeasy Blood and tissue	31.22
HZy_11945	11945	*Hyles zygophylli*	Afghanistan	A.&E.Kotzsch	Entomological Museum, Marktleuthen, Germany	1992	DNeasy Blood and tissue	63.42
HZy_11949	11949	*Hyles zygophylli*	Usbekistan	Eitschberger EMEM	Entomological Museum, Marktleuthen, Germany	1995	DNeasy Blood and tissue	7.00

S1 Table Comprehensive overview for all samples and extractions.

HAp_11951	11951	*Hyles apocyni / hippophaes*	Kirgistan	S.Churkin	Entomological Museum, Marktleuthen, Germany	2000	DNeasy Blood and tissue	9.10
HAp_11952	11952	*Hyles apocyni*	China	Floriani	Entomological Museum, Marktleuthen, Germany	2013	DNeasy Blood and tissue	1659.00
HGa_11953	11953	*Hyles gallii*	China	Grieshuber	Entomological Museum, Marktleuthen, Germany	2007	Monarch PCR&DNA Clean up Kit	199.50
HGa_11954	11954	*Hyles gallii*	Mongolia	Eitschberger EMEM	Entomological Museum, Marktleuthen, Germany	2004	Monarch PCR&DNA Clean up Kit	318.50
HAp_11963	11963	*Hyles apocyni*	Kirgisien	V. Lukhtanov	Entomological Museum, Marktleuthen, Germany	1996	DNeasy Blood and tissue	348.60
HAp_11966	11966	*Hyles apocyni / hippophaes*	Tadjikistan	S.K.Korb	Entomological Museum, Marktleuthen, Germany	2011	innuPREP DNA MIni Kit	570.00
HAp_11967	11967	*Hyles apocyni / hippophaes*	S Tadjikistan	V.Gurko	Entomological Museum, Marktleuthen, Germany	2006	innuPREP DNA MIni Kit	246.00
HAp_11968	11968	*Hyles apocyni / hippophaes*	Kirgistan	S.Churkin	Entomological Museum, Marktleuthen, Germany	2000	innuPREP DNA MIni Kit	588.00
HAp_11969	11969	*Hyles apocyni / hippophaes*	Tadjikistan	V.Gurko	Entomological Museum, Marktleuthen, Germany	2006	innuPREP DNA MIni Kit	222.00
HAp_11972	11972	*Hyles apocyni*	China	Eitschberger EMEM	Entomological Museum, Marktleuthen, Germany	1996	DNeasy Blood and tissue	25.90
HHi_11977	11977	*Hyles hippophaes*	Russia	V. Lukhtanov & A. Dantchenko	Entomological Museum, Marktleuthen, Germany	2001	DNeasy Blood and tissue	44.80
HHi_11978	11978	*Hyles hippophaes*	Kirgisien	V. Lukhtanov	Entomological Museum, Marktleuthen, Germany	1995	DNeasy Blood and tissue	49.56
HHi_11979	11979	*Hyles hippophaes*	Kirgisien	V. Lukhtanov	Entomological Museum, Marktleuthen, Germany	1995	DNeasy Blood and tissue	33.04
HHi_11984	11984	*Hyles hippophaes*	Kasachstan	M.Danilevsky	Entomological Museum, Marktleuthen, Germany	2002	DNeasy Blood and tissue	1127.00
HAp_11989	11989	*Hyles apocyni / hippophaes*	Russia	R. Jakovlev	Entomological Museum, Marktleuthen, Germany	2001	DNeasy Blood and tissue	903.00
HAp_11990	11990	*Hyles apocyni / hippophaes*	Russia	R. Yakovlev	Entomological Museum, Marktleuthen, Germany	2002	DNeasy Blood and tissue	1148.00
HLi_11991	11991	*Hyles livornica*	China	Floriani	Entomological Museum, Marktleuthen, Germany	2013	Monarch PCR&DNA Clean up Kit	98.00
HLi_12091	12091	*Hyles livornica*	Syrie	Rohlena Pavel	Sphingidae museum s.r.o., Příbram, Czech republic	1995	Monarch PCR&DNA Clean up Kit	237.30
HLi_12095	12095	*Hyles livornica*	Pakistan	V. Gurko	Sphingidae museum s.r.o., Příbram, Czech republic	2005	Monarch PCR&DNA Clean up Kit	331.80
HLi_12096	12096	*Hyles livornica*	Pakistan	V. Gurko / Tomás Melichar	Sphingidae museum s.r.o., Příbram, Czech republic	2009	Monarch PCR&DNA Clean up Kit	295.40
HCo_12104	12104	*Hyles costata*	Mongolia	J. Halada	Sphingidae museum s.r.o., Příbram, Czech republic	2005	Monarch PCR&DNA Clean up Kit	168.00
HCo_12105	12105	*Hyles costata*	Mongolia	Odbayar Tz	Sphingidae museum s.r.o., Příbram, Czech republic	2005	Monarch PCR&DNA Clean up Kit	584.50
HCo_12106	12106	*Hyles costata*	Mongolia	Balázs Benedek	Sphingidae museum s.r.o., Příbram, Czech republic	2008	Monarch PCR&DNA Clean up Kit	98.00
HCo_12107	12107	*Hyles costata*	Mongolia	Gurko	Sphingidae museum s.r.o., Příbram, Czech republic	2014	Monarch PCR&DNA Clean up Kit	279.30
HZy_12109	12109	*Hyles zygophylli*	Kasachstan	Sphingidaemuseum,C.R.	Sphingidae museum s.r.o., Příbram, Czech republic	2016	DNeasy Blood and tissue	582.40
HZy_12111	12111	*Hyles zygophylli*	Kasachstan	Sphingidaemuseum,C.R.	Sphingidae museum s.r.o., Příbram, Czech republic	2016	DNeasy Blood and tissue	414.40
HZy_12112	12112	*Hyles zygophylli*	Kasachstan	Sphingidaemuseum,C.R.	Sphingidae museum s.r.o., Příbram, Czech republic	2016	DNeasy Blood and tissue	499.80
HZy_12113	12113	*Hyles zygophylli*	Tadjikistan	Melichar	Sphingidae museum s.r.o., Příbram, Czech republic	2001	DNeasy Blood and tissue	275.80
HZy_12120	12120	*Hyles zygophylli*	Mongolia	Gurko	Sphingidae museum s.r.o., Příbram, Czech republic	2014	DNeasy Blood and tissue	1372.00
HZy_12121	12121	*Hyles zygophylli*	Mongolia	Alois Pavlicko	Sphingidae museum s.r.o., Příbram, Czech republic	2002	DNeasy Blood and tissue	344.40
HNi_12126	12126	*Hyles nicaea*	Syrie	Cadiou	Sphingidae museum s.r.o., Příbram, Czech republic	1932	Monarch PCR&DNA Clean up Kit	994.00
HNi_12127	12127	*Hyles nicaea*	Aserbaijan	I. Pljushtch	Sphingidae museum s.r.o., Příbram, Czech republic	2003	Monarch PCR&DNA Clean up Kit	406.00
HVe_12134	12134	*Hyles vespertilio*	Turkye	-	Sphingidae museum s.r.o., Příbram, Czech republic	1997	Monarch PCR&DNA Clean up Kit	696.50
HVe_12135	12135	*Hyles vespertilio*	Aserbaijan	-	Sphingidae museum s.r.o., Příbram, Czech republic	1986	Monarch PCR&DNA Clean up Kit	952.00
HVe_12136	12136	*Hyles vespertilio*	Georgien	-	Sphingidae museum s.r.o., Příbram, Czech republic	1970	Monarch PCR&DNA Clean up Kit	202.30
HVe_12137	12137	*Hyles vespertilio*	Dalmatien	-	Sphingidae museum s.r.o., Příbram, Czech republic	1938	Monarch PCR&DNA Clean up Kit	595.00
HNi_12139	12139	*Hyles nicaea*	Turkey	J. Krüger	Sphingidae museum s.r.o., Příbram, Czech republic	1984	Monarch PCR&DNA Clean up Kit	584.50
HNi_12142	12142	*Hyles nicaea*	Turkey	György Fábián	Sphingidae museum s.r.o., Příbram, Czech republic	2000	Monarch PCR&DNA Clean up Kit	33.11
HCh_12148	12148	*Hyles chamyla apocyni*	Tajikistan	B.Benedek&S.Ilniczky	Senckenberg Natural History Collections, Dresden, Germany	2017	innuPREP DNA MIni Kit	46.40
HCh_12149	12149	*Hyles chamyla apocyni*	Tajikistan	B.Benedek&S.Ilniczky	Senckenberg Natural History Collections, Dresden, Germany	2017	DNeasy Blood and tissue	424.20
HAp_12151	12151	*Hyles apocyni*	Tadjikistan	V.Gurko	Senckenberg Natural History Collections, Dresden, Germany	2006	DNeasy Blood and tissue	347.20
HSv_12153	12153	*Hyles svetlana?*	Kasachstan	V.Gurko	Senckenberg Natural History Collections, Dresden, Germany	2002	DNeasy Blood and tissue	270.20
HSv_12154	12154	*Hyles svetlana?*	Kasachstan	V.Gurko	Senckenberg Natural History Collections, Dresden, Germany	2002	DNeasy Blood and tissue	142.80
HCe_12155	12155	*Hyles centralasiae*	Tadjikistan	V.Gurko	Senckenberg Natural History Collections, Dresden, Germany	2006	innuPREP DNA MIni Kit	96.00
HCe_12156	12156	*Hyles centralasiae*	Tadjikistan	V.Gurko	Senckenberg Natural History Collections, Dresden, Germany	2006	innuPREP DNA MIni Kit	612.00
HCe_12157	12157	*Hyles centralasiae*	Tadjikistan	V.Gurko	Senckenberg Natural History Collections, Dresden, Germany	2002	innuPREP DNA MIni Kit	540.00
HRo_12159	12159	*Hyles robertsi*	Tadjikistan	V. Gurko	Senckenberg Natural History Collections, Dresden, Germany	1996	DNeasy Blood and tissue	431.20
HAp_12221LT	12221	*Hyles chamyla apocyni*	Tadjikistan	leg. Ju. Shchetkin	Zoological Institute, Saint-Petersburg, Russia	1953	Monarch PCR&DNA Clean up Kit	149.10
HSv_12222HT	12222	*Hyles siehei svetlana*	Kasachstan	Trofimova T.A. & Shovkoon D.	Zoological Institute, Saint-Petersburg, Russia	2006	Monarch PCR&DNA Clean up Kit	302.40
HSv_12223PT	12223	*Hyles siehei svetlana*	Kasachstan	Trofimova T.A. & Shovkoon D.	Zoological Institute, Saint-Petersburg, Russia	2006	Monarch PCR&DNA Clean up Kit	269.85
HCo_12284	12284	*Hyles costata*	Tadjikistan	V.V. Dubatolov	Chinese Academy of Sciences, Institute of Zoology, Beijing, China	1988	Monarch PCR&DNA Clean up Kit	212.10
HEx_12285HT	12285	*Hyles costata exilis*	Tadjikistan	V.V. Dubatolov	Chinese Academy of Sciences, Institute of Zoology, Beijing, China	1982	Monarch PCR&DNA Clean up Kit	486.50
HCo_12387	12387	*Hyles costata*	China	-	Chinese Academy of Sciences, Institute of Zoology, Beijing, China	1959	Monarch PCR&DNA Clean up Kit	465.50
HCo_12389	12389	*Hyles costata*	China	-	Chinese Academy of Sciences, Institute of Zoology, Beijing, China	1973	Monarch PCR&DNA Clean up Kit	451.50
HCo_12390	12390	*Hyles costata*	China	-	Chinese Academy of Sciences, Institute of Zoology, Beijing, China	1962	Monarch PCR&DNA Clean up Kit	201.60
HCo_12392	12392	*Hyles costata*	China	-	Chinese Academy of Sciences, Institute of Zoology, Beijing, China	1972	Monarch PCR&DNA Clean up Kit	106.40
HCo_12396	12396	*Hyles costata*	China	-	Chinese Academy of Sciences, Institute of Zoology, Beijing, China	1980	Monarch PCR&DNA Clean up Kit	353.50
HCo_12399	12399	*Hyles costata*	China	-	Chinese Academy of Sciences, Institute of Zoology, Beijing, China	1981	Monarch PCR&DNA Clean up Kit	514.50
HZy_12400	12400	*Hyles zygophylli*	Pakistan	Major	Collection M. Ströhle, Weiden, Germany	1995	DNeasy Blood and tissue	348.60
HSi_12401	12401	*Hyles siehei*	Tadjikistan	-	Collection M. Ströhle, Weiden, Germany	1990	DNeasy Blood and tissue	397.60
HSi_12403	12403	*Hyles siehei*	Turkmenistan	J. Miatleuski	Collection M. Ströhle, Weiden, Germany	1995	DNeasy Blood and tissue	36.82
HHi_12406	12406	*Hyles hippophaes/ siehei*	Turkmenistan	J. Miatleuski	Collection M. Ströhle, Weiden, Germany	1995	DNeasy Blood and tissue	697.20
HZy_12407	12407	*Hyles zygophylli*	Pakistan	Major	Collection M. Ströhle, Weiden, Germany	1995	DNeasy Blood and tissue	341.60

S1 Table Comprehensive overview for all samples and extractions.

HHi_12409	12409	*Hyles hippophaes*	China	-	Collection M. Ströhle, Weiden, Germany	1996	DNeasy Blood and tissue	67.34
HHi_12410PT	12410	*Hyles hippophaes miatleuskii*	Kasachstan	V. Karalius & I. Miatleuski	Collection M. Ströhle, Weiden, Germany	1999	DNeasy Blood and tissue	506.80
HZy_12411	12411	*Hyles zygophylli*	Kirgistan	V. Dolin	Collection M. Ströhle, Weiden, Germany	1996	DNeasy Blood and tissue	329.00
HHi_12412	12412	*Hyles hippophaes*	China	-	Collection M. Ströhle, Weiden, Germany	1996	DNeasy Blood and tissue	481.60
HHi_12414PT	12414	*Hyles hippophaes miatleuskii*	Kasachstan	V. Karalius & I. Miatleuski	Collection M. Ströhle, Weiden, Germany	1999	DNeasy Blood and tissue	592.20
HHi_12415	12415	*Hyles hippophaes/ siehei*	Turkmenistan	J. Miatleuski	Collection M. Ströhle, Weiden, Germany	1995	DNeasy Blood and tissue	45.36
HSi_12418	12418	*Hyles siehei*	Türkei	P. Kautt & V. Weisz	Collection M. Ströhle, Weiden, Germany	1997	DNeasy Blood and tissue	511.00
HEu_12420	12420	*Hyles euphorbiae*	Türkei	H. Thöny	Collection M. Ströhle, Weiden, Germany	1985	DNeasy Blood and tissue	595.00
HZy_12421	12421	*Hyles zygophylli*	China	coll. Ströhle	Collection M. Ströhle, Weiden, Germany	1996	DNeasy Blood and tissue	555.80
HSi_12422	12422	*Hyles siehei*	Usbekistan	Veisz	Collection M. Ströhle, Weiden, Germany	1992	DNeasy Blood and tissue	403.20
HZy_12423	12423	*Hyles zygophylli*	Iran?	Czipka	Collection M. Ströhle, Weiden, Germany	1975	DNeasy Blood and tissue	84.00
HEu_12424	12424	*Hyles euphorbiae*	Usbekistan	Veisz	Collection M. Ströhle, Weiden, Germany	1992	DNeasy Blood and tissue	621.60
HSi_12425	12425	*Hyles siehei*	Türkei	P. Kautt & V. Weisz	Collection M. Ströhle, Weiden, Germany	1997	DNeasy Blood and tissue	483.00
HEu_12428	12428	*Hyles euphorbiae*	Armenien	Dantschenko	Collection M. Ströhle, Weiden, Germany	2001	Monarch PCR&DNA Clean up Kit	307.30
HHi_12436	12436	*Hyles hippophaes / apocyni*	Turkmenistan	ӡy. Fabian, B. Herczig, A. Podlussany & Z. Varga	Museum Witt, München, Germany	1992	DNeasy Blood and tissue	144.20
HHi_12438	12438	*Hyles hippopaes*	Indien	P. Kautt & V. Weisz	Museum Witt, München, Germany	1994	DNeasy Blood and tissue	791.00
HHi_12439	12439	*Hyles hippophaes*	Indien	P. Kautt & V. Weisz	Museum Witt, München, Germany	1994	DNeasy Blood and tissue	763.00
HHi_12441	12441	*Hyles hippophaes*	China	-	Museum Witt, München, Germany	1996	DNeasy Blood and tissue	243.60
HCe_12450	12450	*Hyles centralasiae*	Turkmenistan	ӡy. Fabian, B. Herczig, A. Podlussany & Z. Varga	Museum Witt, München, Germany	1992	DNeasy Blood and tissue	126.70
HCe_12451	12451	*Hyles centralasiae*	Turkmenistan	ӡy. Fabian, B. Herczig, A. Podlussany & Z. Varga	Museum Witt, München, Germany	1992	DNeasy Blood and tissue	38.64
HEu_12492	12492	*Hyles euphorbiae*	Aserbaijan	Museum Witt via Roman Yakovlev	Museum Witt, München, Germany	1974	Monarch PCR&DNA Clean up Kit	511.00
HEu_12497	12497	*Hyles euphorbiae*	Turkmenistan	ӡy. Fabian, B. Herczig, A. Podlussany & Z. Varga	Museum Witt, München, Germany	1992	DNeasy Blood and tissue	491.40
HEu_12499	12499	*Hyles euphorbiae*	Usbekistan	Museum Witt via Roman Yakovlev	Museum Witt, München, Germany	1994	DNeasy Blood and tissue	330.40
HZy_12521	12521	*Hyles zygophylli*	Iran	Szabó & Hentschel	Museum Witt, München, Germany	2000	DNeasy Blood and tissue	298.20
HCo_12551	12551	*Hyles costata*	China	Wang Ying	Chinese Academy of Sciences, Institute of Zoology, Beijing, China	1960	Monarch PCR&DNA Clean up Kit	202.30
HGa_12565	12565	*Hyles gallii nepalensis*	India	leg. P. Kautt & V. Weisz	Entomological Museum, Marktleuthen, Germany	15.07.1994	Monarch PCR&DNA Clean up Kit	539.00
HEx_12566HT	12566	*Hyles chuvilini*	Russia	leg. Chuvilin A.V.	Entomological Museum, Marktleuthen, Germany	20.07.1993	Monarch PCR&DNA Clean up Kit	654.50
HEx_12567AT	12567	*Hyles chuvilini*	Russia	leg. Chuvilin A.V.	Entomological Museum, Marktleuthen, Germany	20.07.1993	Monarch PCR&DNA Clean up Kit	658.00
HEx_12568PT	12568	*Hyles chuvilini*	China	ex coll. Franz Eichler, Wittenberg	Entomological Museum, Marktleuthen, Germany	in ENEM 26.02.1992	Monarch PCR&DNA Clean up Kit	668.50
HEx_12570	12570	*Hyles chuvilini*	Mongolia	leg. Aidas Saldaitis	Entomological Museum, Marktleuthen, Germany	11.06.2004	Monarch PCR&DNA Clean up Kit	364.00
HEx_12572	12572	*Hyles exilis*	Mongolia	leg. A. Saldaitis	Entomological Museum, Marktleuthen, Germany	27-30.06.2003	Monarch PCR&DNA Clean up Kit	226.80
HEx_12573	12573	*Hyles exilis*	Mongolia	leg. A. Saldaitis	Entomological Museum, Marktleuthen, Germany	14-15.06.2003	Monarch PCR&DNA Clean up Kit	277.20
HEx_12574	12574	*Hyles exilis*	Mongolia	leg. Odbayar Tz	Entomological Museum, Marktleuthen, Germany	14.06.2005	Monarch PCR&DNA Clean up Kit	353.50
HEx_12575	12575	*Hyles exilis*	Siberia	leg. Kruger, Saldaitis	Entomological Museum, Marktleuthen, Germany	01-04.08.2000	Monarch PCR&DNA Clean up Kit	274.40
HEx_12576	12576	*Hyles exilis*	Mongolia	leg. Odbayar Tz.	Entomological Museum, Marktleuthen, Germany	14.06.2005	Monarch PCR&DNA Clean up Kit	511.00
HSt_12580PT	12580	*Hyles stroehlei*	Pakistan	lgt. Z. Weidenhoffer	Entomological Museum, Marktleuthen, Germany	27.06.1992	Monarch PCR&DNA Clean up Kit	1106.00
HSt_12581PT	12581	*Hyles stroehlei*	Pakistan	leg. M. Hreblay & G. Csorba	Entomological Museum, Marktleuthen, Germany	21.06.1992	Monarch PCR&DNA Clean up Kit	68.60
HSt_12582PT	12582	*Hyles stroehlei*	Pakistan	leg. B. Herczig, Gy. M. Lászlo & G. Ronkay	Entomological Museum, Marktleuthen, Germany	12.07.1994	Monarch PCR&DNA Clean up Kit	63.70
Hhi_12622LT	12622	*Hyles hippophaes*	Romania	-	Hessisches Landesmuseum für Kunst und Natur, Wiesbaden, Germany	[1789]	Monarch PCR&DNA Clean up Kit	350.00
Hve_12623LT	12623	*Hyles vespertillio*	Italy	-	Hessisches Landesmuseum für Kunst und Natur, Wiesbaden, Germany	[1780]	Monarch PCR&DNA Clean up Kit	679.00
Hli_12624LT	12624	*Hyles livornica*	Italy	-	Hessisches Landesmuseum für Kunst und Natur, Wiesbaden, Germany	[1779]	Monarch PCR&DNA Clean up Kit	749.00
HLi_12625T	12625	*Hyles livornica*	Italy	-	Hessisches Landesmuseum für Kunst und Natur, Wiesbaden, Germany	[1779]	Monarch PCR&DNA Clean up Kit	73.50
HLi_12626T	12626	*Hyles livornica*	Italy	-	Hessisches Landesmuseum für Kunst und Natur, Wiesbaden, Germany	[1779]	Monarch PCR&DNA Clean up Kit	263.20
