# Supplementary material for: Advantages of an easy-to-use DNA extraction method for minimal-destructive analysis of collection specimens
Source: PLoS One. 2020 Jul 8;15(7):e0235222. doi: 10.1371/journal.pone.0235222 (PMC7343169; doi:10.1371/journal.pone.0235222)
Supplement: S2 Table — (PDF) [file pone.0235222.s004.pdf]

S2 Table. Extracted DNA amounts from six specimens of *Hyles chamyla* and *Hyles svetlana* comparing the DNeasy and the Monarch protocol.

MTD-TW Accession No.	Species	Location	Age (*estimated)	Total extracted DNA (ng)	
				DNeasy	Monarch
9248	*H. chamyla*	Kirgistan, Naryn	131 *	13.9	251.7
9251	*H. chamyla*	Turkmenistan, Mary	111 *	37.6	345.0
9252	*H. chamyla*	Turkmenistan, Mary	111 *	28.2	303.0
9353	*H. chamyla*	China, Xinjiang Kumul	111 *	17.6	387.0
9254	*H. chamyla*	unknown	unknown	13.5	354.0
9255	*H. svetlana*	Kasachstan, Qysylorda	2006	0.0	603.0
