# Supplementary material for: Advantages of an easy-to-use DNA extraction method for minimal-destructive analysis of collection specimens
Source: PLoS One. 2020 Jul 8;15(7):e0235222. doi: 10.1371/journal.pone.0235222 (PMC7343169; doi:10.1371/journal.pone.0235222)
Supplement: S3 Table — (PDF) [file pone.0235222.s005.pdf]

S4 Table. Shotgun libraries of 10 specimens, identity of sequences were assigned using Kraken2.

MTD-TW Accession No.	Species	Age (*estimated)	No. read pairs	Reads mapped to *Hyles* (%)	Reads mapped to bacteria (%)	Reads mapped to fungi (%)	Reads mapped to human (%)	Unclassified reads (%)
9228	*Hyles centralasiae*	22	785,913	60.05	1.1	0.38	0.44	38.03
9248	*Hyles chamyla*	131 *	2,004,453	38.96	2.44	0.59	2.95	55.06
9254	*Hyles chamyla*	unknown	706,016	30.58	3.28	1.74	1.48	62.92
12566	*Hyles exilis*	25	2,562,187	45.49	0.71	0.23	0.28	53.29
12567	*Hyles exilis*	25	2,066,378	55.81	0.64	0.22	0.24	43.09
12622	*Hyles hippophaes*	231 *	1,912,671	9.21	0.85	0.34	8.81	80.79
12624	*Hyles livornica*	241 *	2,083,280	37.27	0.83	0.67	2.57	58.66
9233	*Hyles siehei*	unknown	1,086,100	29.91	1.52	0.43	0.68	67.46
9255	*Hyles svetlana*	12	2,373,991	49.44	0.36	0.63	1.27	48.3
12623	*Hyles vespertilio*	240 *	2,221,361	60.04	0.39	0.71	2.45	36.41
