# Supplementary material for: Advantages of an easy-to-use DNA extraction method for minimal-destructive analysis of collection specimens
Source: PLoS One. 2020 Jul 8;15(7):e0235222. doi: 10.1371/journal.pone.0235222 (PMC7343169; doi:10.1371/journal.pone.0235222)
Supplement: S4 Table — (PDF) [file pone.0235222.s006.pdf]

S5 Table. Coverage summary for alignments of the shotgun libraries of 10 specimens.

MTD-TW Accession No.	Non-zero (bp)	Zero (bp)	Non-zero (%)	Zero (%)
9228	15309	0	100.00	0.00
9248	15045	264	98.28	1.72
9254	14408	901	94.11	5.89
12566	15295	14	99.91	0.09
12567	15288	21	99.86	0.14
12622	13873	1436	90.62	9.38
12624	15309	0	100.00	0.00
9233	14869	440	97.13	2.87
9255	15245	64	99.58	0.42
12623	15309	0	100.00	0.00
**Mean**	**14995**	**314**	**97.95**	**2.05**
**SD**	**± 367**	**± 367**	**± 2.40**	**± 2.40**
